# Molecular dynamics simulations of the adsorption of an intrinsically disordered protein: Force field and water model evaluation in comparison with experiments

**DOI:** 10.3389/fmolb.2022.958175

**Published:** 2022-10-26

**Authors:** Mona Koder Hamid, Linda K. Månsson, Viktoriia Meklesh, Per Persson, Marie Skepö

**Affiliations:** ^1^ Division of Theoretical Chemistry, Lund University, Lund, Sweden; ^2^ Centre for Environmental and Climate Science, Lund University, Lund, Sweden; ^3^ Lund Institute of Advanced Neutron and X-ray Science (LINXS), Lund, Sweden

**Keywords:** intrinsically disordered proteins (IDPs), molecular dynamics, force field (FF), water models, adsorption, conformational ensemble

## Abstract

This study investigates possible structural changes of an intrinsically disordered protein (IDP) when it adsorbs to a solid surface. Experiments on IDPs primarily result in ensemble averages due to their high dynamics. Therefore, molecular dynamics (MD) simulations are crucial for obtaining more detailed information on the atomistic and molecular levels. An evaluation of seven different force field and water model combinations have been applied: (A) CHARMM36IDPSFF + CHARMM-modified TIP3P, (B) CHARMM36IDPSFF + TIP4P-D, (C) CHARMM36m + CHARMM-modified TIP3P, (D) AMBER99SB-ILDN + TIP3P, (E) AMBER99SB-ILDN + TIP4P-D, (F) AMBERff03ws + TIP4P/2005, and (G) AMBER99SB-disp + disp-water. The results have been qualitatively compared with those of small-angle X-ray scattering, synchrotron radiation circular dichroism spectroscopy, and attenuated total reflectance Fourier transform infrared spectroscopy. The model IDP corresponds to the first 33 amino acids of the N-terminal of the magnesium transporter A (MgtA) and is denoted as KEIF. With a net charge of +3, KEIF is found to adsorb to the anionic synthetic clay mineral Laponite^®^ due to the increase in entropy from the concomitant release of counterions from the surface. The experimental results show that the peptide is largely disordered with a random coil conformation, whereas the helical content (α- and/or 3_10_-helices) increased upon adsorption. MD simulations corroborate these findings and further reveal an increase in polyproline II helices and an extension of the peptide conformation in the adsorbed state. In addition, the simulations provided atomistic resolution of the adsorbed ensemble of structures, where the arginine residues had a high propensity to form hydrogen bonds with the surface. Simulations B, E, and G showed significantly better agreement with experiments than the other simulations. Particularly noteworthy is the discovery that B and E with TIP4P-D water had superior performance to their corresponding simulations A and D with TIP3P-type water. Thus, this study shows the importance of the water model when simulating IDPs and has also provided an insight into the structural changes of surface-active IDPs induced by adsorption, which may play an important role in their function.

## 1 Introduction

Intrinsically disordered proteins (IDPs) lack a singular equilibrium structure. Instead, they sample a heterogeneous ensemble of largely disordered conformations with only temporary secondary structures (Dunker et al., 2001; Tompa, 2002; Uversky and Dunker, 2010). Due to this, IDPs are challenging to study experimentally, where usually only ensemble averages can be investigated. Hence, simulations are valuable for receiving information at the atomistic and molecular levels. There are some force fields explicitly developed for IDPs, but it is a relatively new field where it is not evident which force field is optimal for a given system.

Moreover, IDPs may acquire a varying degree of structure depending on their environment, which makes it unclear what force field to use in scenarios where IDPs may obtain more ordered characteristics. In this study, we investigate seven combinations of force fields and water models for the IDP we refer to as KEIF, both in solution and adsorbed to a surface, where we have indications from prior studies that significant altercations occur in its secondary structure upon adsorption (Jephthah et al., 2020). It was shown that KEIF adsorbs to the surface of anionic large unilamellar vesicles (LUVs), with a concomitant structural change, resulting in an increased α-helical structure. Adsorption was presumed to be electrostatic due to the opposite charge of the peptide and the vesicles and entropically driven by the release of counterions.

KEIF represents the first 33 amino acids of the N-terminal of the magnesium transporter A (MgtA), a protein found in bacteria that serves as a primary active transporter pump for magnesium ions across the cell membrane (Maguire, 2006). KEIF was identified as intrinsically disordered in a recent study by Subramani et al. (2016), using the DISOPRED3 server for intrinsically disordered region (IDR) prediction (Jones and Cozzetto, 2015). The primary sequence of KEIF is as follows:

MFKEIFTRLIRHLPSRLVHRDPLPGAQQTVNTV.

At physiological pH, KEIF carries five positively charged (blue) and two negatively charged (red) amino acids, giving it a net charge of +3. Most charged amino acids are evenly distributed in the first half of the peptide. Of the positively charged residues, one is lysine, and the remaining are arginine residues. Specific for arginine is its guanidinium group with three hydrogen donor sites.

A solid surface, synthetic clay mineral Laponite^®^ was chosen for its simplicity to investigate the adsorbed state of KEIF as an initial study toward a better understanding of its adsorption mechanism and the structural changes that follow. Laponite^®^ particles are disc-shaped with a radius of 25 nm and thickness of 1 nm. Laponite^®^ belongs to the 2:1 clay mineral and consists of an octahedral magnesium oxide layer sandwiched between two tetrahedral silica layers. Partial substitution of Mg^2+^ with Li^+^ gives the surface a net negative charge. The rim, however, has a positive charge from the protonation of exposed hydroxyl groups. Studies on the interactions between Laponite^®^ and various therapeutic biomolecules have been of interest in recent years due to its prospect as a drug delivery vehicle (Koshy et al., 2018; Gaharwar et al., 2019; Zheng et al., 2019). One such biomolecule class is the cationic antimicrobial peptides, of which many are IDPs (Yacoub et al., 2017; Zsila et al., 2018; Haffner et al., 2019). Hence, studying the surface adsorption of IDPs to Laponite^®^ is also of interest from a medical aspect.

Here, we use the following seven force fields and water models to study KEIF: (A) CHARMM36IDPSFF (Liu et al., 2018) + CHARMM-modified TIP3P (MacKerell et al, 1998), (B) CHARMM36IDPSFF + TIP4P-D (Piana et al., 2015), (C) CHARMM36m (Huang et al., 2017) + CHARMM-modified TIP3P, (D) AMBER99SB-ILDN (Lindorff-Larsen et al., 2010) + TIP3P (Jorgensen et al., 1983), (E) AMBER99SB-ILDN + TIP4P-D, (F) AMBERff03ws (Best et al., 2014, Piana et al., 2015) + TIP4P/2005 (Abascal and Vega, 2005), and (G) AMBER99SB-disp + disp-water (Piana et al., 2015, Robustelli et al., 2018, Piana et al., 2020). These are compared with experiments using small-angle X-ray scattering (SAXS), synchrotron circular dichroism spectroscopy (SRCD), and attenuated total reflectance Fourier transform infrared spectroscopy (ATR-FTIR, from now on denoted as IR) to obtain information about the conformation and structure of KEIF.

This study aims to gain an insight into which force field and water combinations that are appropriate for studying surface-active IDPs, both in solution, where they are primarily disordered, and in their adsorbed state, where they may obtain a more ordered structure. In addition, we would like to better understand the adsorption mechanism and the subsequent conformational and structural changes.

## 2 Materials and methods

### 2.1 Materials

Synthetic KEIF (3.871 kDa) of purity 95.67% was bought from Genemed Synthesis Inc. (San Antonio, United States). To remove impurities, the peptide was dissolved in and dialyzed (100–500 Da MWCO Biotech Cellulose Ester (CE) Dialysis Membrane Tubing, Spectrum Labs, Piraeus, Greece) against Milli-Q water. After that, the sample was freeze-dried to obtain the purified peptide.

Synthetic Laponite^®^ (Laponite®-XLG XR) (BYK, 2016) clay mineral with a cation exchange capacity of 0.5 mEq/g, a specific surface area of 370 m/g^2^ (Brunauer–Emmett–Teller, BET (Brunauer et al., 1938)), a density of 2,530 kg/cm^3^, a radius of 25 nm, and a thickness of 1 nm was bought from BYK Additives & Instruments (Wesel, Germany) and was used without further purification.

### 2.2 Methods: Experiments

#### 2.2.1 Synchrotron radiation circular dichroism spectroscopy

SRCD was used to probe the secondary structural changes induced by KEIF’s adsorption to Laponite^®^. A volume of 1.0 mg/ml KEIF was mixed with a Laponite^®^ dispersion of 1.6 mg/ml in 20 mM phosphate buffer prepared by mixing sodium phosphate monobasic monohydrate (0.0049 M) and sodium phosphate dibasic dihydrate (0.01508 M), with pH set to 7.4, and the final pH in the sample was set to 7.6. The ionic strength was set to 10 mM by NaF. The SRCD measurements were performed at the AU-CD beam line on the synchrotron light source, ASTRID2, at the Department of Physics and Astronomy, Aarhus University, Denmark, with a standard setup. Hence, the samples were measured in a 0.1-mm quartz cuvette, and the spectra were recorded between 180 and 270 nm at 20°C. The obtained SRCD spectra were subject to BeStSel (Micsonai et al., 2015; Micsonai et al., 2018) fitting through a web server, http://bestsel.elte.hu/index.php , to access the corresponding secondary structure elements. BeStSel uses secondary structure basis components derived from DSSP (Kabsch and Sander, 1983), and it is possible to detect α-helices, β-strands, and hydrogen-bonded turns, which include hydrogen-bonded β-turns. Fitted residuals are presented in Supplementary Figure S1.

#### 2.2.2 Infrared spectroscopy

The secondary structure was also studied using IR. Measurements were performed using a VERTEX 80v FTIR spectrometer (Bruker, Ettlingen, Germany) equipped with an attenuated total reflectance accessory (FastIR, Harrick Scientific, NY, United States). A modified version of the simultaneous infrared and potentiometric titration method was used by Loring et al. (2009). A ZnSe crystal was assembled in a titration vessel and mounted in the ATR accessory. The bottom of the vessel (optical part) was kept under a vacuum. Samples were kept at room temperature, under constant stirring and continuous nitrogen flow during measurements. Separate adsorption experiments were performed at KEIF concentrations of 0.21, 0.31, and 0.49 mg/ml of 20 mM tris(hydroxymethyl)aminomethane (TRIS), pH 7.4, and 10 mM NaCl. The pH was maintained at 7.4 using a burette system 907 Titrando (Metrohm AG, Herisau, Switzerland) with the regulated addition of 39.0 mM HCl or 41.99 mM NaOH.

A volume of 10 mg/ml solution of KEIF in the absence of Laponite^®^ was measured using a transmission IR accessory with a liquid cell. It is to be noted that we could not use the ATR accessory with ZnSe or diamond crystals, which usually gives a better signal-to-noise ratio than a conventional transmission cell, because of the peptide adsorbed on the surface of the crystals. KEIF and the buffer solution were measured separately by confining 25 μL of the sample between CaF_2_ windows (diameter of 25 mm). The spectrometer was continuously purged with CO_2_-free dry air to remove water vapors. Built-in OPUS 7.2 software (Bruker, Billerica, United States) for atmospheric compensation was applied to remove residual water vapors. A total of 128 scans/spectra were recorded. Subtraction of buffer was achieved by changing the subtraction factor until a straight baseline was obtained between 2,500 cm^−1^ and 1,700 cm^−1^.

The IR spectra of KEIF were cut between 1,110 and 1,750 cm^−1^ and baseline-corrected using an asymmetric least square method. The secondary structure was inferred from the analysis of the amide I band in the IR spectrum 1,700–1,600 cm^−1^. To resolve separate secondary structure components, a second derivative analysis was performed on the amide I band, resulting in four peaks corresponding to β-strand at 1,620 cm-1 and 1,638 cm^−1^, random coils/α-helices at 1,654 cm^−1^, and β-turns at 1,678 cm^−1^ (Singh, 1999). The amide I band was fitted, assuming a Gaussian shape of each peak with a bandwidth of 30 cm^−1^. The relative amount of each structural component was calculated from the integrated area under each peak. The algorithm in our analyses was similar to the one described by Singh (1999) and Arrondo et al. (1993). The details of fitting are given in Supplementary Section S2. All processing of the spectra was performed using OPUS 7.2 software.

### 2.3 Methods: Simulations

#### 2.3.1 Atomistic MD simulations

Atomistic MD simulations were performed using the GROMACS package version 2021 (Berendsen et al., 1995; Abraham et al., 2015). Simulations of KEIF were performed for the single chain in solution and adsorbed to a Laponite^®^ surface. Seven different force field and water model combinations were applied for the peptide, as shown in Table 1, along with CLAYFF (Cygan et al., 2004) for Laponite^®^.

**TABLE 1:** Force fields and water models used for simulations and the labels used to denote them.

Label	Force field	Water model
A	CHARMM36IDPSFF	CHARMM-modified TIP3P
B	CHARMM36IDPSFF	TIP4P-D
C	CHARMM36m	CHARMM-modified TIP3P
D	AMBER99SB-ILDN	TIP3P
E	AMBER99SB-ILDN	TIP4P-D
F	AMBERFF03WS	TIP4P/2005
G	AMBER99SB-disp	Disp-water

An initial linear configuration of KEIF was constructed with Avogadro (an open-source molecular builder and visualization tool), version 1.2.0, available at Hanwell et al. (2012). The zwitterionic state of the N- and the C-termini was used. The charge of the side chains was set at physiological pH, giving KEIF a net charge of +3. A dodecahedron simulation box under periodic boundary conditions (PBCs) in all directions was used for KEIF in solution with a minimum distance of 10 Å between KEIF and the box edges. A cubic box (131 × 136 × 70 Å^3^) was used for KEIF with Laponite^®^ under PBC in all directions. The initial linear structure of KEIF was positioned with the backbone in parallel to the surface and midway between the surface and its periodic image along the *z*-coordinate. The Laponite^®^ surface was obtained by replicating 25 × 15 times the unit cell. Each unit cell had a charge of -1, thus giving the surface a charge density of -0.021 *e*/Å^3^ (for full details on the unit cell, see Supplementary Section S3). Chloride or sodium counterions were added for neutrality for KEIF in solution or with Laponite^®^, respectively.

The equations of motion were integrated using the Verlet leap-frog algorithm (Berendsen and Van Gunsteren, 1986) with a time step of 2 fs. A Verlet list cut-off scheme was used for the non-bonded interactions, and short-ranged interactions were calculated using a pair list with a cut-off of 12 Å, for all force field and water model combinations. Long-ranged dispersion corrections were applied to the systems’ energy and pressure. Long-ranged electrostatics was calculated using smooth particle-mesh Ewald method (Darden et al., 1993) with cubic interpolation and a grid spacing of 1.6 Å. Bond lengths involving hydrogen were constrained using LINCS algorithm (Hess et al., 1997). The temperature was set to 298 K using a velocity-rescaling thermostat (Bussi et al., 2007), with a relaxation time of 0.1 ps, and a separate coupling group was used for KEIF. Isotropic pressure coupling was achieved using a Berendsen barostat (Berendsen et al., 1984) to maintain a pressure of 1.0 bar, with a relaxation time of 2.0 ps and isothermal compressibility of 4.5 × 10^−5^ bar^−1^, corresponding to that of water.

Energy minimization was achieved with the steepest-descent algorithm. The system was equilibrated for 0.5 ns in the NVT ensemble, followed by 1.0 ns in the NPT ensemble, with position restraints applied to KEIF in both steps. Position restraints were lifted for the 1.0 μs production run in the NVT ensemble. Six replicates were performed with different starting seeds to improve sampling. Replicates deemed as outliers where the peptide became fixed in one conformation were removed; the details are shown in the Supplementary Material. The same settings were used for the peptide both in solution and with Laponite^®^, except that a slower PBC algorithm was used for the latter due to the periodicity of the surface. For simulations, D-G of AMBER-type force fields, Laponite^®^ frozen to circumvent the different settings between them, and CLAYFF for generating non-specified pairwise parameters for non-bonded interactions of the AMBER-type force fields were used.

#### 2.3.2 Simulation analyses

All simulation analyses were performed on the concatenated trajectory of the five replicates, achieved with the GROMACS tool “gmx trjconv.” Calculation of radius of gyration, *R_g_* was carried out using “gmx polystat.” Conversion to distributions was performed using an in-house code. Theoretical scattering intensities were calculated using CRYSOL, version 3.0.3 (Svergun et al., 1995). The pair distance distribution, P(r), was computed using PRIMUS, version 3.0.3 (Konarev et al., 2003). The secondary structure content was determined with the DSSPPII program, which is the modified version by Chebrek et al. (2014) of the DSSP program Kabsch and Sander, 1983 that includes the determination of left-handed polyproline II (PPII) helices. The PPII helix is an extended structure with a helical pitch of 9.3 Å/turn. In addition to PPII helices, the DSSPPII algorithm also detects α-helices, 3_10_-helices, β-strands, β-bridges, hydrogen-bonded turns, and random coils (Kabsch and Sander, 1983). Ramachandran plots were obtained using the GROMACS tool “gmx rama.” The minimum distance and the number of hydrogen bonds between KEIF and Laponite^®^ were estimated using “gmx pairdist” and “gmx hbond,” respectively. Clusters were found using the “gmx cluster” tool, using the GROMOS method (Daura et al., 1999) and with a root-mean-square deviation cut-off distance of 8.0 Å. Visualization of structures was achieved using VMD (version 1.9.4) (Humphrey et al., 1996).

## 3 Results and discussion

Seven different force field and water model combinations were used to study KEIF in solution and adsorbed to a Laponite^®^ surface using MD simulations, and the results were compared with those of SAXS, SRCD, and IR experiments. First, we present the results of KEIF in solution, second, in the adsorbed state, and last, compare the two results. We refer to the seven different force field and water model combinations: (A) CHARMM36IDPSFF + CHARMM-modified TIP3P, (B) CHARMM36IDPSFF + TIP4P-D, (C) CHARMM36m + CHARMM-modified TIP3P, (D) AMBER99SB-ILDN + TIP3P, (E) AMBER99SB-ILDN + TIP4P-D, (F) AMBERff03ws + TIP4P/2005, and (G) AMBER99SB-disp + disp-water.

### 3.1 KEIF in solution

#### 3.1.1 Theoretical and experimental scattering data

**FIGURE 1:**
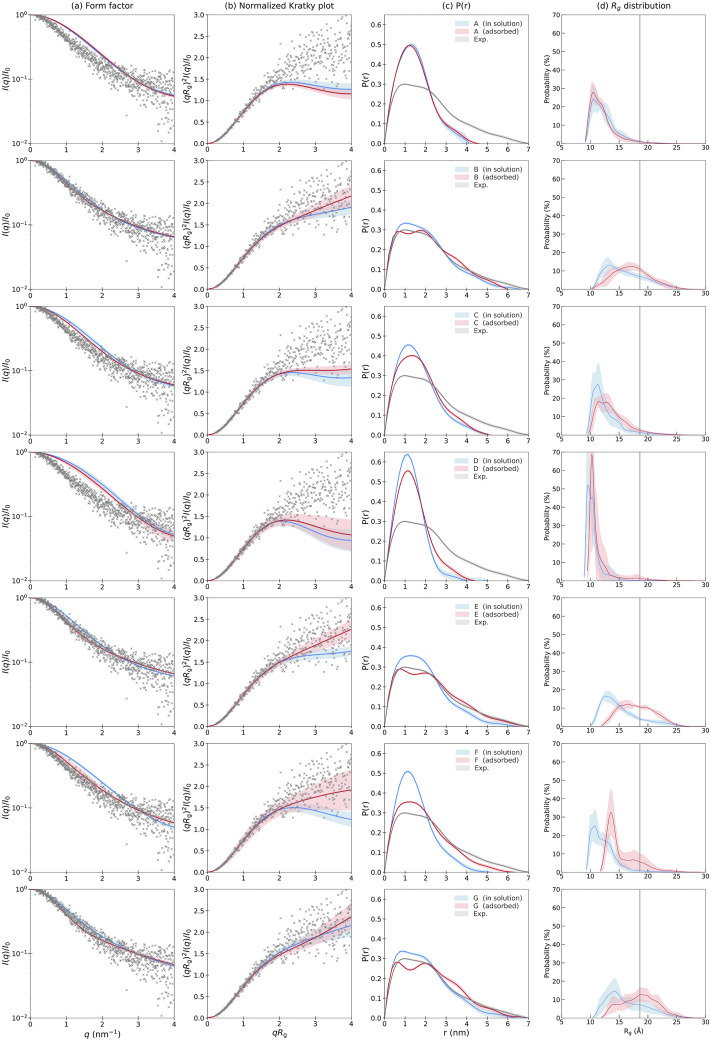
**(A)** Form factor, **(B)** normalized Kratky plot, **(C)** pair distance distribution, and **(D)** radius of gyration distribution for KEIF in solution (blue) and adsorbed (red) for the different force field and water model combinations, with shaded areas representing the standard deviation between replicates. Experimental SAXS results are included for KEIF in solution (gray) as described by Jephthah et al. (2020).

In Figure 1, theoretical scattering curves (blue) from the MD simulations are compared to experimental SAXS data (gray) collected in a prior study by Jephthah et al. (2020). Simulations B, E, and G agreed better with the experimental scattering curve than the simulations of the other systems. The experimentally normalized Kratky plot indicates a random coil, which is in line with the results obtained from B, E, and G, whereas A, C, D, and F indicate a more compact protein with a globular shape. The experimental P(r), shows a broad distribution extending up to 6 nm, with an overall maximum of 1–2 nm. Simulations B, E, and G similarly display broad distributions with peak values at 1–2 nm. They are thus in good agreement with the experimental data, however, having somewhat narrower distributions and a lower maximum intra-particle distance. Simulations A, C, D, and F have significantly more limited distributions, extending up to approximately 4–5 nm and with distinct peaks at roughly 1.2 nm. In Figure 1D, the *R_g_*, and distribution for the simulations are shown and compared with the average experimental *R*_*g*_ (gray line) as determined from Guinier analysis. It is seen that all simulations underestimate *R*_*g*_; however, while simulations A, C, D, and F do so severely, the broad distributions of B, E, and G do sample the experimentally determined *R*_*g*_ to some extent. It is notable that simulations B and E, using the TIP4P-D water model, are more consistent with experiments than their analogues A and D, using TIP3P-type water. Among B, E, and G, it is observed that G has a slightly better experimental agreement.

#### 3.1.2 Secondary structural analysis

##### 3.1.2.1 Experimental results

**FIGURE 2:**
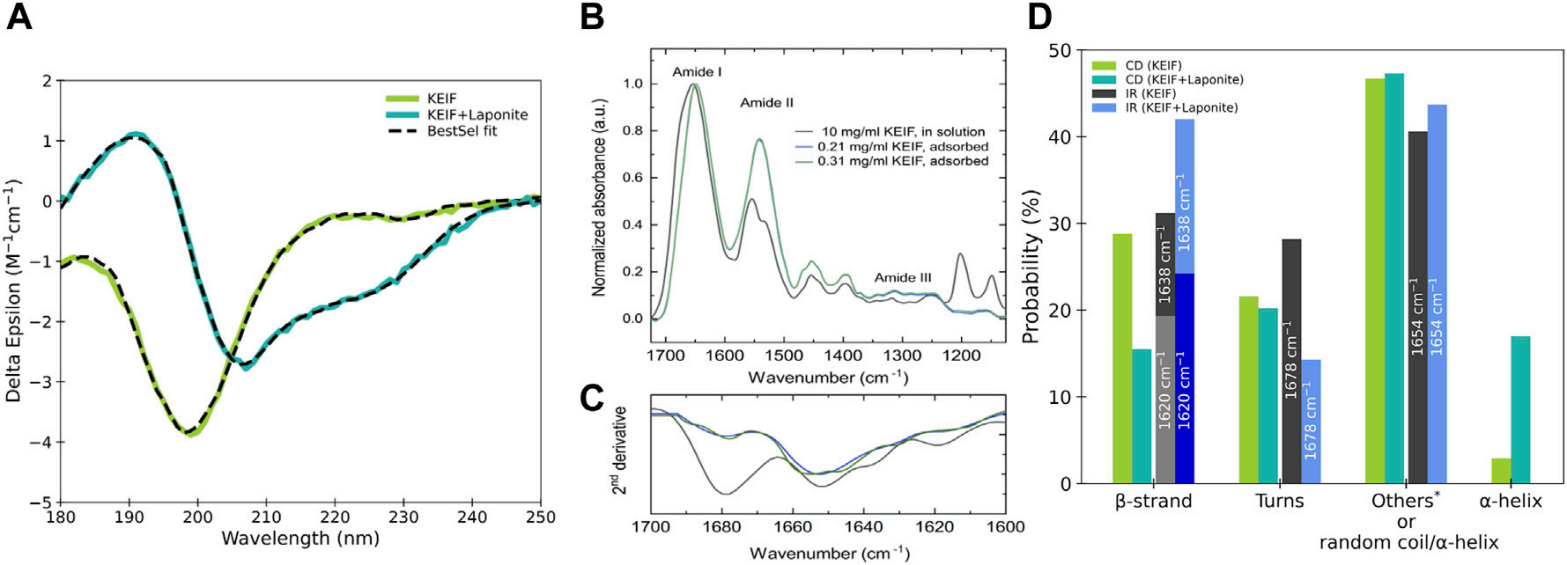
Experimental data of KEIF in solution and with Laponite^®^. **(A)** SRCD data, **(B)** ATR-FTIR data, and **(C)** their second derivative analysis. **(D)** Probability of secondary structure elements for KEIF was obtained from the fitting by BeStSel. Structures within the DSSP classification undetermined by BeStSel fitting of SRCD are included in “others”: 3_10_-helix, bends, and random coil.

The secondary structure of KEIF in solution was examined experimentally using SRCD and IR, as presented in Figure 2. The SRCD spectrum shown for KEIF in solution is characteristic of a disordered structure with a strong negative band below 200 nm (Chemes et al., 2012). By fitting the spectrum with BeStSel, it is observed that undetermined (other) structures constitute the most significant portion, likely attributed to the peptides’ disordered structure, which agrees with the random coil conformation obtained from SAXS. The fit also indicated a considerable fraction of β-strands and a small portion of α-helices. PPII helices are not included among the secondary structure basis components of BeStSel but are of interest here as they are more common for IDPs than previously believed (Jephthah et al., 2021). They are identified from SRCD by a strong negative band at approximately 198 nm and a weak positive band at approximately 218 nm (Chemes et al., 2012). Here, the SRCD spectra have a strong negative band near 198 nm and a weak band near 218 nm, which can be attributed to the presence of PPII helices.

From IR, the secondary structure content of KEIF was determined from the curve-fitting of the amide I signal. Second-derivative analysis was used to define the position and the number of bands. The IR spectrum of KEIF exhibits characteristic amide bands, typically for peptides with helical and β-strand structures (Dong et al., 1990; Arrondo et al., 1993; Singh, 1999). The second-derivative analysis identified four bands at 1,620 nm, 1,638 nm, 1,654 nm, and 1,678 nm. According to Singh (1999), the bands at 1,620 nm and 1,638 nm arise from β-strands, whereas the band at 1,654 nm can be assigned to α-helices and/or random coils. Thus, we also analyzed the amide III region to distinguish between the two components (Singh, 1999). However, both structures were found in the amide III region, as shown in Supplementary Figure S4. In addition, it should be noted that side chain vibrations can also give rise to signals in this region (Singh, 1999). Finally, the band at 1,678 nm is attributed to β-turns, the most common type of turns. The relative amount of the four identified bands is shown in Figure 2D. The estimated proportion of secondary structures for IR is in reasonably good agreement with that from SRCD. Both offer a high presence of β-strands and -turns. IR does not distinguish between random coils and α-helices. Although by considering the low proportion of α-helices from SRCD, it is probable that the 1,654 nm band arises mainly from a random coil, which would be consistent with the high disorder observed with SRCD and SAXS and is expected for IDPs.

The estimated secondary structure content from SRCD and IR should be considered cautiously since accurate fitting can be challenging. For SRCD, the BeStSel tool was used, which for some peptides has been seen to overestimate β-strands (Miles et al., 2021). In addition, the analysis is sensitive to the precise peptide concentration, and hence, any possible inaccuracies in the concentration affect the quality of the analysis (Contreras-Martos et al., 2018). For IR, accurate background subtraction is critical as the buffer absorbs in the amide I region, which is used for secondary structure determination. Furthermore, measurements are sensitive to residual water vapors, which are strongly absorbed in the amide I region (Natalello et al., 2012).

Moreover, it is worth noting for both techniques that similar structures may be indistinguishable; signals from β-strands and α-helices may be due to β-bridges and 3_10_-helices, respectively. Hence, while simulations may differentiate between related structures, this may not be the case for the experiments conducted. Henceforth, when comparing simulations to experiments, the sum of β-bridges and β-strands will be referred to as β-content, and the sum of α- and 3_10_-helices will be referred to as helical content.

#### 3.1.3 Molecular dynamics simulations

**FIGURE 3:**
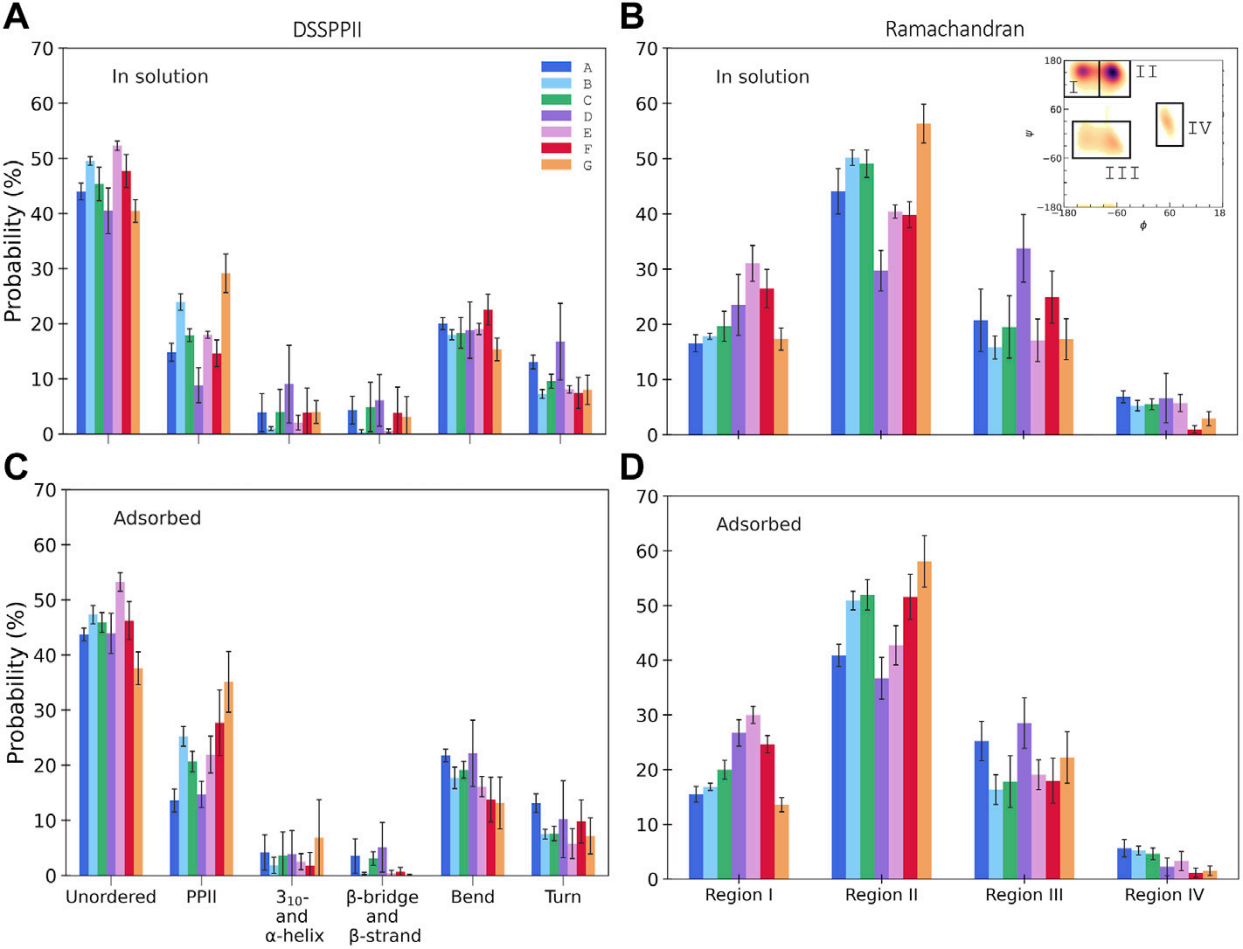
Secondary structure content from integrated Ramachandran plots, in the regions as indicated in the inset (see the Supplementary Material for specified angles), for KEIF in solution **(A)** and adsorbed **(B)**, and DSSPPII analysis for KEIF in solution **(C)** and adsorbed **(D)**. Structures known to occur in the Ramachandran regions are I) β-strands, II) PPII helices, III) 3_10_- and right-handed α-helices, and IV) left-handed α-helices. Error bars represent the standard deviation between replicates.

DSSPPII analysis of the MD simulations was performed, which calculates the hydrogen bonding pattern from which the average secondary structure could be estimated, as shown in Figure 3A. All simulations observe a high degree of disorder, which is in agreement with the largely disordered system obtained by experiments. In addition, all yield a relatively high degree of PPII helices. Interestingly, simulations B and E, with TIP4P-D water, display a much higher PPII content than their analogues A and D, with TIP3P-type water. Simulation G had the most significant proportion of PPII helices of 30.6 ± 4.6%. All simulations give a low helical content, as expected from SRCD. Neither simulations have much β-content, contrary to experiments. One reason experiments show higher β-content than simulations could be because simulations only consider a single peptide in contrast to experiments, where it is possible that peptides associate and aid the formation of β-strands as they can be stabilized by β-sheet formation between β-strands of different peptides. It is less likely for β-sheets to form in a single peptide due to its short sequence (Nowick, 2008). The proportion of turns in the simulations varies between 7 and 16%, less than that observed by experiments. In addition, DSSPPII measures a significant presence of bends, which is registered neither by SRCD nor by IR.

The secondary structure in the simulations was also investigated by Ramachandran analysis, shown in Figure 3B. Four regions were identified that include, but not exclusively, the following structures: (I) β-strands, (II) PPII helices, (III) 3_10_- and right-handed α-helices, and (IV) left-handed α-helices, with dihedral angles at approximately (φ, ψ) of (I) (-135°, 135°), (II) (-75°, 145°), (III) (-50°, -30°) and (-60°, -45°), and (IV) (60°, 35°). The regions were integrated to obtain estimates of each structure (for initial Ramachandran plots, please see Supplementary Figures S42, S43). All simulations have a high count in the PPII region. Again, B and E observed higher PPII content than A and D, respectively; G had the highest PPII content. The degree of β-strands from Ramachandran analysis was significantly higher than that estimated by DSSPPII. Simulations D, E, and F had a more significant proportion of β-strands than the remainder. In addition, the Ramachandran analysis also showed notable counts in regions (III) and (IV), which may be attributed to helical content. However, these signals may be obtained from other structures with similar dihedral angles, for example, turns.

### 3.2 KEIF adsorbed to Laponite^®^ compared to that in solution

#### 3.2.1 The peptide conformation

Theoretical scattering curves characterized the peptide conformation in its adsorbed state, normalized Kratky plots, and P(r) curves, as shown in Figure 1 (red curves). For simulations A and D, the normalized Kratky plots remain as a globular and compact structure. In contrast, simulation F is now indicative of a random coil, and simulation C is in between that of a random coil and a globular conformation. The normalized Kratky plots of simulations B, E, and G show an extended random coil conformation. For all simulations except A, the normalized Kratky plots suggest that the peptide becomes more extended when adsorbed to the Laponite^®^ surface, with the most drastic changes notable for simulation F, followed by E, B, and G. These results are reflected by the broadening of P(r) in the adsorbed state for all simulations except A. Simulation F again experiences the largest broadening and extension of the maximum distance from 5 to 6 nm. Simulations B, E, and G also observed broadening of their distributions with an apparent double peak at approximately 0.5 and 2.0 nm, implying that two conformations of comparable probability exist. Similarly, the *R_g_* distribution in Figure 1D is shifted toward larger values for simulations B, E, F, and G, while no evident changes of the distributions are observed for A, C, and D.

From the results of the theoretical scattering, we again notice significant discrepancies between simulations differing only by their water model. Moreover, those with the same (B and E) or similar water models (A, C, and D) produce comparable results. Hence, the choice of a water model is essential for predicting the conformation, both for the peptide in solution and the adsorbed state.

#### 3.2.2 Structural analysis

SRCD and IR experiments were also performed for KEIF with Laponite^®^, and secondary structure components were estimated from their fitting, as shown in Figure 2. Comparing KEIF adsorbed to Laponite^®^ with KEIF in solution, a significant increase in α-helices is observed for SRCD. The 1,654 nm band increases for IR, indicating that the combined amount of random coils and α-helices increases. SRCD shows that the structure remains highly disordered. IR has an increase in β-strands during these decreases for SRCD.

From DSSPPII analysis, as shown in Figure 3C, all simulations show that the peptide in the adsorbed state remains highly disordered, where simulation E predicts the highest degree of disorder. All simulations also offer a high content of PPII helices, the highest for simulation G, which appear to increase upon adsorption for all simulations except A, and most significantly for F. Moreover, simulations B and E again show higher PPII content than their analogues A and D, respectively, and C shows a value in between. Similar trends are observed for region II of the Ramachandran analysis, where PPII helices are found, as shown in Figure 3D. From DSSPPII analysis, the probability of bends and turns is comparable to that of KEIF in solution. All simulations have shallow β-content and helical content according to the DSSPPII analysis. However, the integrated Ramachandran plots suggest a more significant presence of β-strands in region I and the helical content found in parts of regions III and IV. Of note, as previously mentioned, the regions do not exclusively correspond to these structures.

### 3.3 Interactions between KEIF and Laponite^®^

#### 3.3.1 Differences in surface interactions along the peptide sequence

**FIGURE 4:**
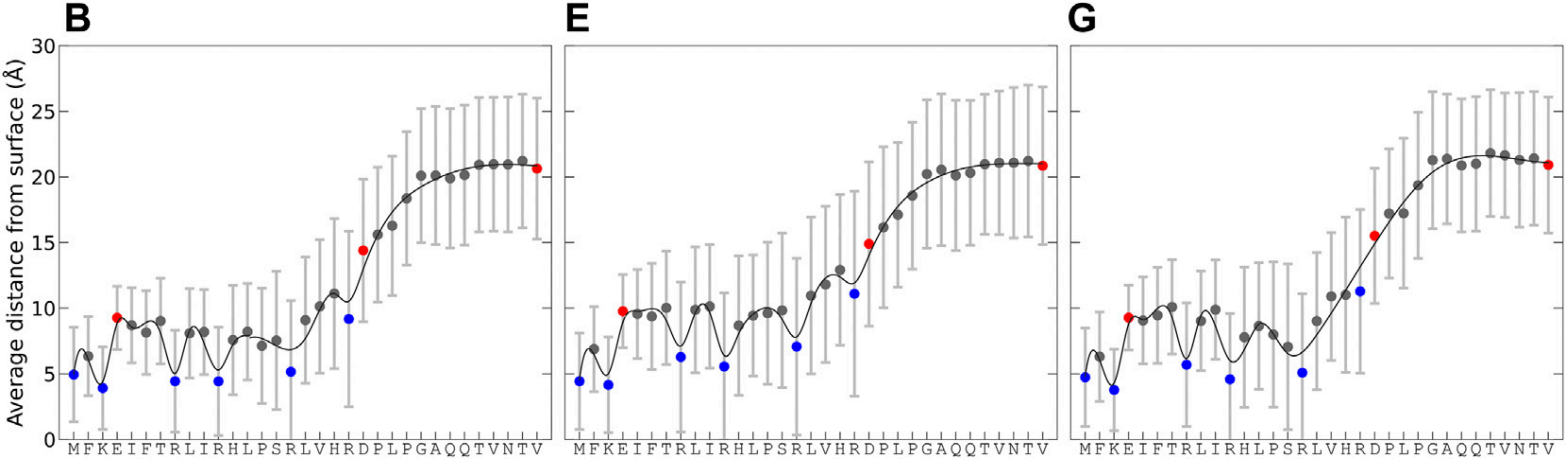
Average distance from the Laponite® surface for each residue and its standard deviation, calculated from the change in minimum distance between each pair over time for the concatenated trajectory of the replicates. For the force field and water combinations B, E, and G. Charge of residues indicated with the colors: blue (positive), red (negative), and gray (neutral). Lines are included as a “guide to the eye.”

Simulations B, E, and G had the best agreement with experimental data for KEIF in solution, and comparable conformational and structural characteristics in the adsorbed state were selected to examine how the surface interactions vary along the peptide sequence. Figure 4 shows the average distance of residues to the surface over time for the concatenated simulation trajectory and its standard deviation, which describes the average distance fluctuations and thus gives an insight into the dynamics. The average distance is the lowest for the first 16 residues, between 0 and 15 Å, and then it increases for residues 17 to 24, before leveling out for the last residues, 25 to 33 to a distance between 15 and 27 Å. This trend is observed in all simulations. The shorter distance to the surface for the first half of the peptide is not unexpected as they include the positively charged N-terminal of methionine, lysine, and arginine that all have an electrostatic attraction to the negative surface. Thus, the adsorption of KEIF to Laponite^®^ is electrostatic and is likely entropically driven by the concomitant release of counterions from the surface. The second half of the peptide has a net electrostatic repulsion to the surface, with one positive arginine and one negative aspartic acid at neighboring positions 20 and 21, followed by neutral residues and the negative C-terminal at the end. Without any net electrostatic attraction to the surface, adsorption of the second half of the peptide is not entropically favorable as the restriction of the peptide’s motion cannot be compensated by the release of counterions. The standard deviation for the average distance further supports this explanation. Comparing the simulations, they observe similar trends; however, simulation E has slightly higher fluctuations, and the first half of the peptide is further away from the surface than B and G.

**FIGURE 5:**
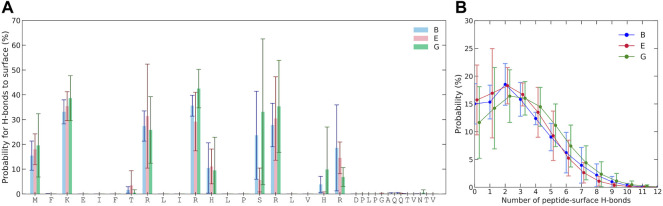
**(A)** Probability of multiple hydrogen bonds between KEIF and Laponite® during the simulations and the probability of at least one hydrogen bond is included in the legend. **(B)** Probability for each residue to the hydrogen bond. Results are shown for the force field and water combinations B, E, and G. Error bars represent the standard deviation between replicates.

The probability for each residue to hydrogen bond with the Laponite^®^ surface was also examined, as shown in Figure 5A. For hydrogen bonding to occur with the surface, the amino acid must have donor hydrogen and follow the criteria for hydrogen bonding, which is a minimum of 3.5 Å donor–acceptor distance and restrictions on the permitted angle. Negligible hydrogen bonding occurs for residues 21 to 33, as they lack any prominent hydrogen donors and remain too distant from the surface. A relatively high hydrogen-bonding probability occurs for the amino group of the N-terminal and that of lysine. Arginines, with their guanidino group, also have a high propensity for hydrogen bonding. Serine, with its hydroxyl group, also shows a possibility for significant hydrogen bonding, although varying between simulations and their replicates. Some degree of hydrogen bonding with the surface is also found for histidines and threonine. Due to several viable hydrogen donors, multiple hydrogen bonds between KEIF and Laponite^®^ are possible, as can be seen in Figure 5B, where there is even some probability of 10 hydrogen bonds at any given instance.

Figure 4 and Figure 5A suggest that the first 20 amino acid residues of KEIF from the N-terminal have a stronger interaction with the negatively charged Laponite^®^ than the remainder. In the context of KEIF being the intrinsically disordered N-terminal region of the more significant protein MgtA, this result implies that the first 20 residues of KEIF will strongly interact with the anionic head groups of a lipid bilayer. Thus, it is reasonable to suspect that this segment will be inserted into the outer leaflet of the bilayer. The end segment of KEIF, which connects to the remainder of the protein, will instead remain outside the bilayer. This observation is in line with the hypothesis established by Jephthah et al. (2020) that the role of KEIF is to anchor the protein to a cell membrane through electrostatic interactions with anionic head groups.

#### 3.3.2 Structural changes along the sequence

**FIGURE 6:**
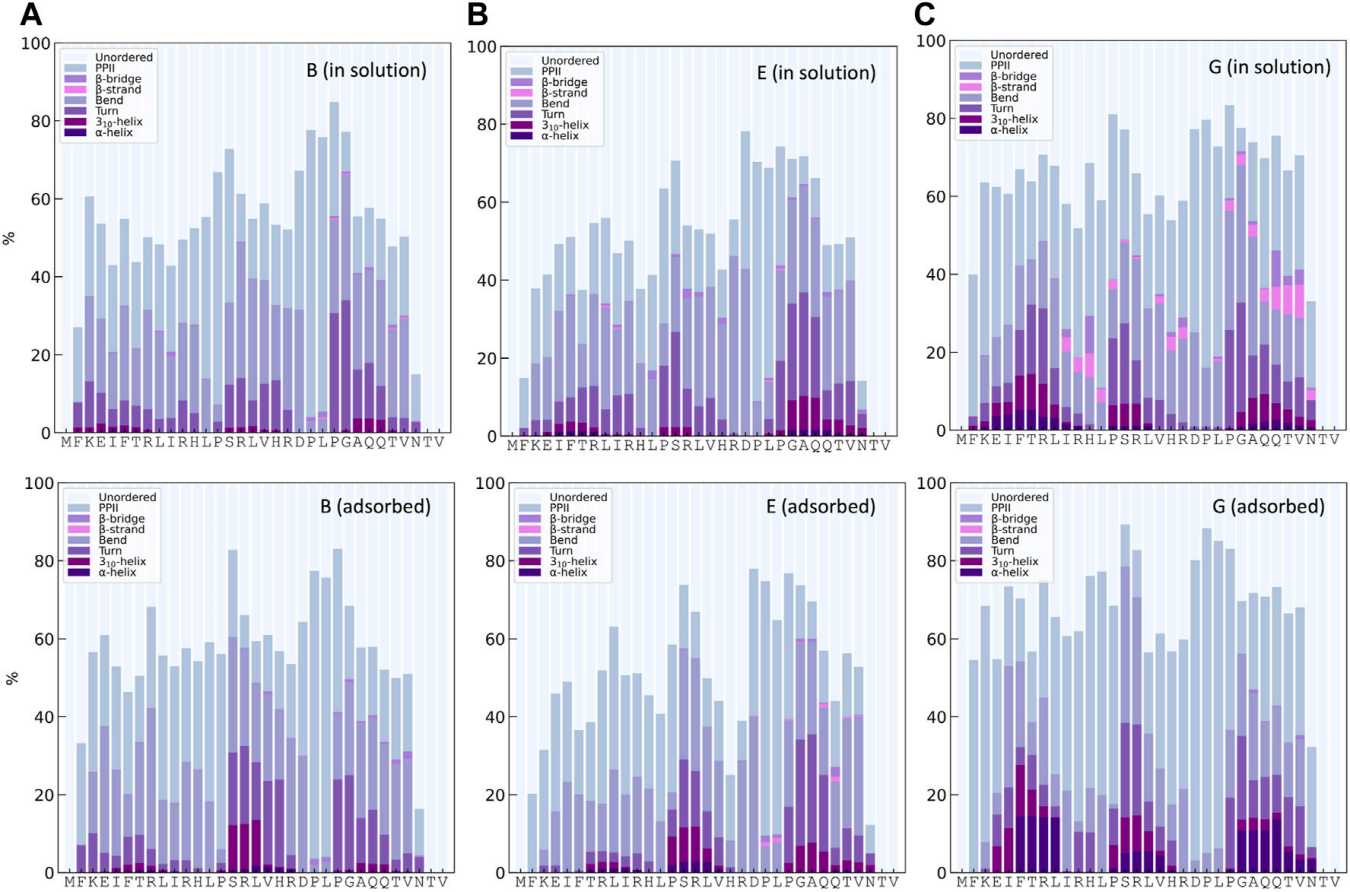
Secondary structure content along the sequence from DSSPPII analysis for KEIF in solution (top) and adsorbed (bottom) for the force field andwater combinations B, E, and G.

The distribution of secondary structures along the peptide sequence for simulations B, E, and G, both for KEIF in solution and adsorbed, is presented in Figure 6. In common for simulations, B and E is approximately 10% 3_10_- and α-helices for the mid-segment of the peptide in its adsorbed state compared to when in solution. Simulation G obtains helical structures in the mid-segment both for the peptide in solution and adsorbed, with an increase in the adsorbed state. In addition, simulation G shows some helical structure at the beginning and the end of the peptide. Simulation E has some helical structure at the end of the sequence. Although the average helical content across all residues, as shown in Figure 3, is low, the helical content for specific residues is significantly higher. Simulation G also has some β-strands and β-bridges in the peptide, which seem to disappear for the adsorbed state.

#### 3.3.3 Closer inspection of the mid-segment of KEIF

The structure of the mid-segment of KEIF and its interactions with the surface was examined further, as three simulations, B, E, and G all suggest that it has high interaction with the surface and seems to obtain increased helical structure.

**FIGURE 7:**
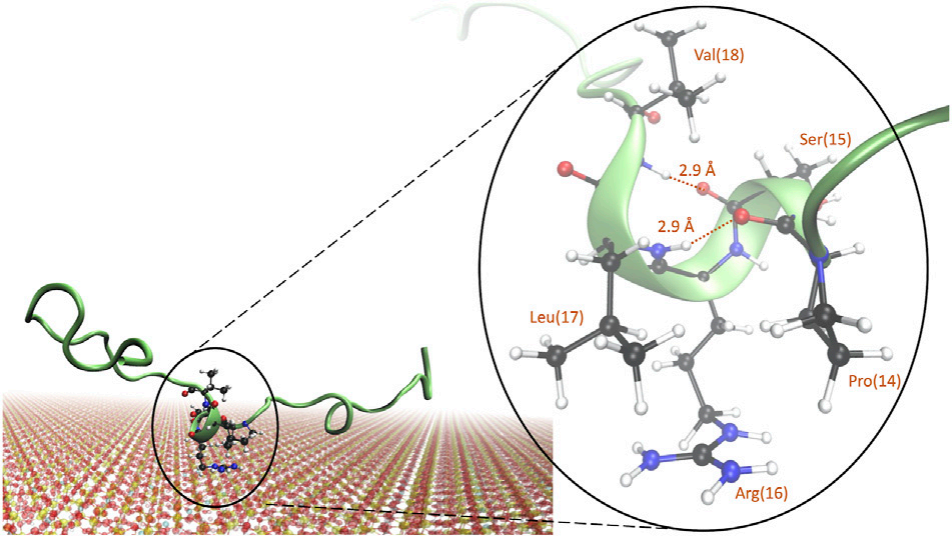
Representative image of a commonly occurring structure in the adsorbed state of KEIF with a 3_10_-helix present for the “SRL” sequence. Atoms are shown for the “PSRLV” sequence, with the hydrogen bonds and distances marked.

From cluster analysis of simulations B, E, and G, we could notice that the formation of a 3_10_-helix in the “SRL” segment, corresponding to residues 15, 16, and 17, was indeed common, occurring in approximately 20% of the clusters. A representative image of the structure is shown in Figure 7. Closer examination reveals that the 3_10_-helix is obtained from both hydrogen bonds between proline-14 and leucine-17 and serine-15 and valine-18, with a hydrogen bond length of 2.9 Å for both. Arginine-16 is positioned close to the surface, which is in line with the observations from Figures 4, Figure 5B. We hypothesize that when arginine-16 adsorbs to the surface, the atoms obtain an orientation that favors hydrogen bonding between near residues and enables the formation of a 3_10_-helix.

## 4 Conclusion

This study investigated the force field and water model choice for MD simulations of a surface-active IDP. We studied its conformational and structural characteristics in solution and adsorbed states and compared them with experiments. Seven different force field and water model combinations were used: (A) CHARMM36IDPSFF + CHARMM-modified TIP3P, (B) CHARMM36IDPSFF + TIP4P-D, (C) CHARMM36m + CHARMM-modified TIP3P, (D) AMBER99SB-ILDN + TIP3P, (E) AMBER99SB-ILDN + TIP4P-D, (F) AMBERff03ws + TIP4P, and (G) AMBER99SB-disp + disp-water. The results indicate that the choice of a water model seems of particular importance, where both CHARMM36IDPSFF and AMBER99SB-ILDN were in better agreement with experimental scattering data when combined with TIP4P-D water rather than CHARMM-modified TIP3P or TIP3P, respectively. The preference for the unconventional combination of CHARMM36IDPSFF with TIP4P-D rather than CHARMM-modified TIP3P is noteworthy. As observed from the SAXS and SRCD experiments, a random coil conformation is expected. Both simulations, B and E, using TIP4P-D, had a random coil conformation, while their analogues A and D, using three-point water, had a globular conformation. Simulation G was also in good agreement with experiments, while C and F were not. Hence, simulations B, E, and G had the best experimental agreement.

Structural content was experimentally studied using SRCD and IR and theoretically studied from DSSPPII and Ramachandran analysis of the simulations. Experiments indicated a highly disordered structure, as expected of IDPs and the simulations, with varying degrees. Simulations B and E were more disordered than their analogues A and D. Despite the high level of disorder, SRCD and IR also showed a significant proportion of β-strands and some presence of α-helices for KEIF in solution. These results were not reflected by the simulations, which vastly underestimated the presence of these secondary structural elements. The discrepancy is possibly due to simulations only considering the single peptide or may indicate a shortcoming in describing IDPs’ force field and water model combinations.

Further investigations are required for clarification. In addition, the experimental data’s fitting procedure has limitations and may not detect all types of structural elements. For example, the SRCD curve has bands indicative of PPII helices, which cannot be captured in the data fitting procedure. Simulations also suggest a high degree of PPII helices, with simulation G predicting the highest proportion. Simulations B and E have higher PPII content than their analogues A and D. Experiments indicate a significant increase in α-helices for KEIF adsorbed to Laponite^®^ compared to that in solution. Simulations do not show any considerable increase, on average, for the entire peptide sequence; however, some increase in 3_10_- and α-helices is observed for specific residues when adsorbed. In common for simulations B, E, and G were the increase in 3_10_- and α-helices in the mid-segment of KEIF, which also had a high interaction with the Laponite^®^ surface through hydrogen bonding. Arginines are often hydrogen-bonded with the surface, and it is hypothesized that it favors the formation of helices. Moreover, simulations revealed an extension of the peptide when adsorbed. The first half of the peptide binds at the surface due to its electrostatic attraction, while the second half extends outwards, allowing greater freedom of movement and an entropy increase.

This study has shown that the choice between the force field and water model is not straightforward when simulating IDPs. In addition to choosing a force field adapted for IDPs, the combined water model has a substantial influence. We have also demonstrated the advantages of using MD simulations with experiments when studying IDPs. Most experimental techniques measure ensemble averages, while simulations allow detailed information with atomistic resolution. From this preliminary study of the adsorption of IDPs, we receive an insight into the role of residues in the adsorption and the resulting conformational and structural changes.

## Data Availability

The raw data supporting the conclusion of this article will be made available by the authors, without undue reservation.
