# Supplementary material for: Molecular dynamics simulations of the adsorption of an intrinsically disordered protein: Force field and water model evaluation in comparison with experiments
Source: Front Mol Biosci. 2022 Oct 26;9:958175. doi: 10.3389/fmolb.2022.958175 (PMC9644065; doi:10.3389/fmolb.2022.958175)
Supplement: Supplementary file 1 [file DataSheet1.pdf]

Supplementary Material: Molecular dynamics simulations of the adsorption of an intrinsically disordered protein: force field and water model evaluation in comparison with experiments

Mona Koder Hamid [1], Linda K. Månsson [1], Viktoriia Meklesh [2], Per Persson [2], and Marie Skepö[1,3*]

[1] Division of Theoretical Chemistry, Lund University, P.O. Box 124, SE-221 00 Lund, Sweden

[2] Centre for Environmental and Climate Science, Lund University, Sölvegatan 37, P.O. Box 188, SE-221 00 Lund, Sweden

[3] LINXS Institute of Advanced Neutron and X-ray Science, Scheelevägen 19, SE-223 70 Lund, Sweden

***Correspondence:**

Marie Skepö

marie.skepo@teokem.lu.se

1 Residuals from BeStSel fitting of CD spectra

Some of the obtained CD spectra were subject to BeStSel (Micsonai et al., 2018, Micsonai et al., 2015) fitting to access KEIF's corresponding secondary structure elements. Fitted residuals are given in Figure S1 and represent the goodness of the fittings that are graphically displayed in Figure 2a in the paper.

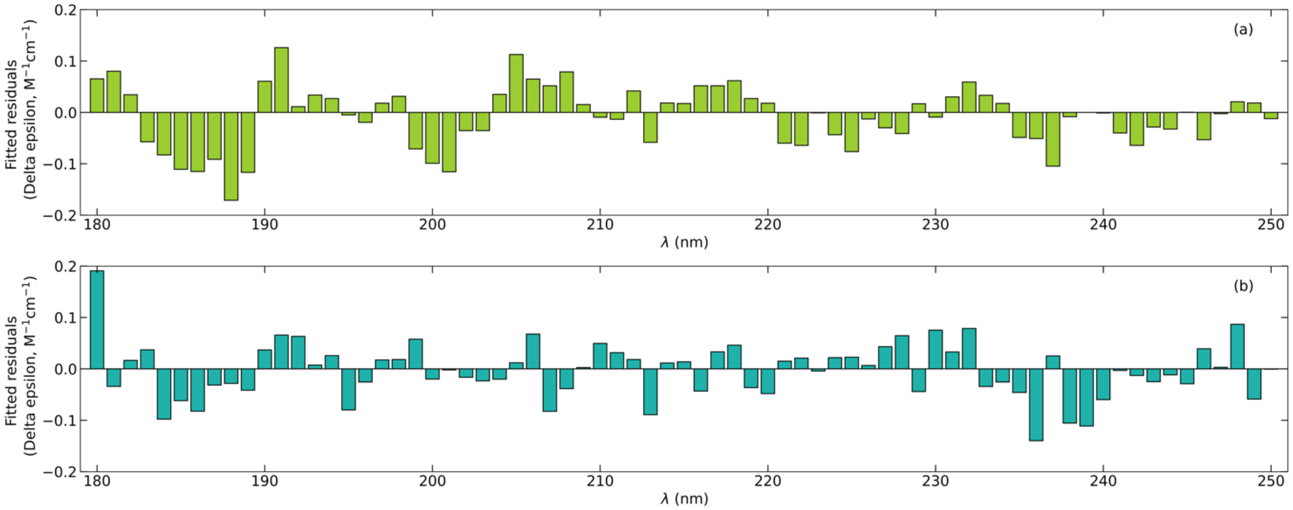

Supplementary Figure 1. Fitted residuals using BeStSel for SRCD spectra of (a) 1 mg/ml KEIF and (b) 1 mg/ml KEIF + 1.6 mg/mL Laponite® both in a phosphate buffer at pH 7.6, 10 mM NaF. The The fits are graphically presented in Figure 1a in the paper.

2 IR spectroscopy

2.1 Experimental details

To make a film of Laponite® 300 μL 2.0 g/L clay suspension was dried on the ATR crystal, already mounted in the sample vessel. The film was equilibrated with 35 mL Laponite® suspension (0.2 g/L, 10 mM NaCl) and monitored for 24 h. When no change was observed in the IR spectra a background spectrum was collected with 1024 scans/spectrum with a resolution of 4 cm^{-1}. Adsorption experiments

were initiated by adding 5mL of KEIF solution to the vessel. KEIF was dissolved in 10mM NaCl and preadjusted to pH 7.4. Adsorption spectra were collected every minute (64 scans/spectrum for the first hour, then every 7 minutes for 512 scans/spectrum) up until 24 h. Desorption experiments were initiated by removing the suspension (note, that around 1 ml of liquid remained in the vessel in order to keep the Laponite® film hydrated) and replacing with a freshly prepared Laponite® suspension (0.2 g/L, 10 mM NaCl). Desorption was monitored for 24 h. (512 scans/spectrum). Figure S2 shows the resulting spectra and how they evolve over time.

2.2 Fitting details

The deconvolution algorithm assumed a Gaussian shape and described as follows (Singh, 1999, Arrondo et al., 1993):

1. Frequencies were fixed at the values detected by the second derivative analysis. Full-width at high-height (FWHH) was fixed to 30 cm-1. Peak intensities were adjusted manually to fit the Amide I and Amide II spectral shape. (Amide II was also included in the fit to avoid a systematic error that may be caused by forcing the band at 1600 cm- 1 to baseline level).
2. Only intensities were iterated (using Levenberg-Marquardt algorithm in OPUS 7.2 software).
3. Only widths were iterated.
4. Only frequencies are iterated.

This loop (2-4) was repeated until the root-mean-square deviation was minimized and close to 0. See the results of the fitting in Figure S3.

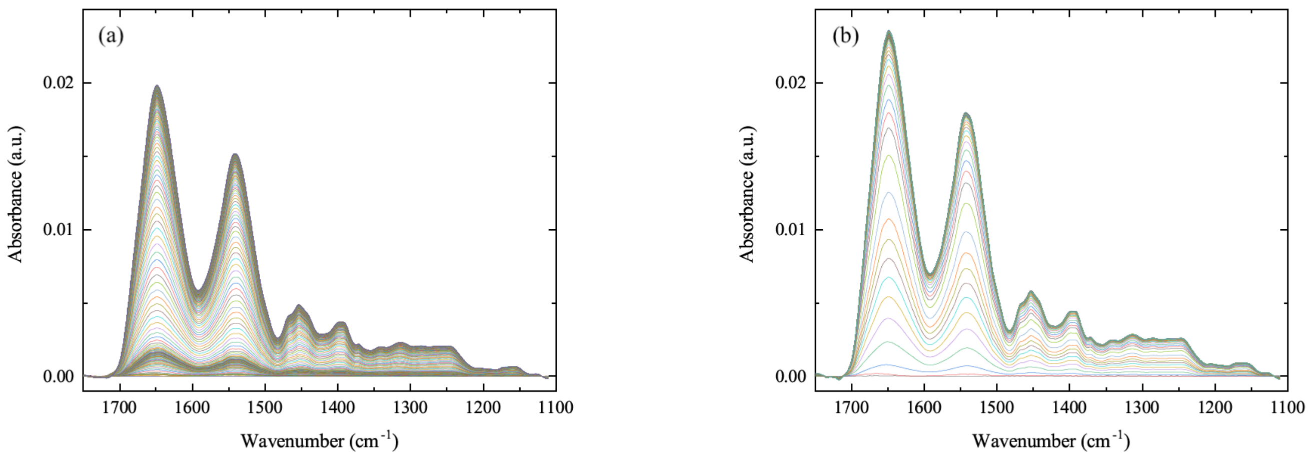

Supplementary Figure 2. IR spectra of KEIF adsorbed on Laponite® at pH 7.4 measured during 24h. Concentrations of peptide are (a) 0.21 mg/mL and (b) 0.31 mg/mL with 10 mM NaCl.

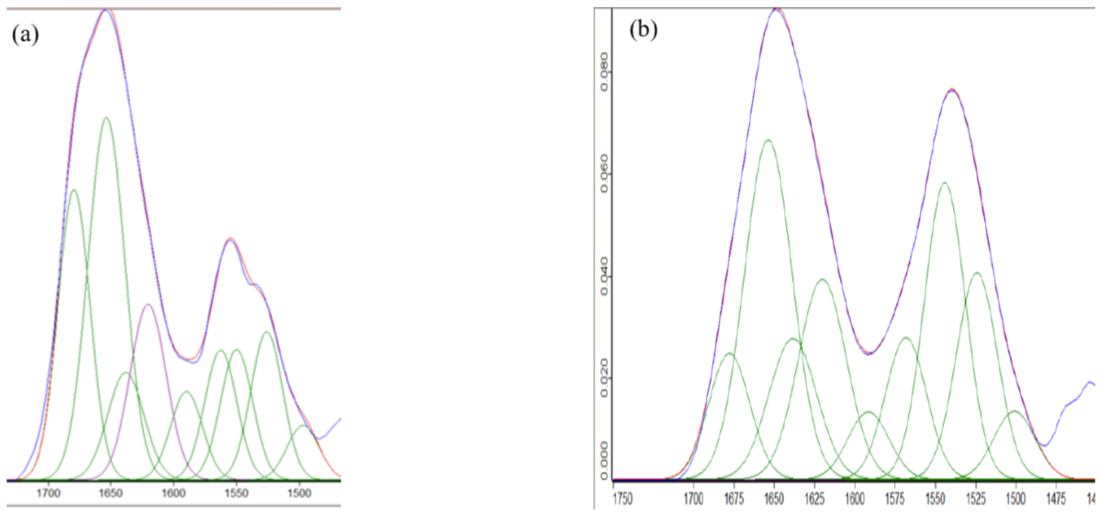

Supplementary Figure 3. Curve-fitted IR spectrum of (a) 10 mg/mL KEIF in-solution and (b) 0.31 mg/mL KEIF adsorbed to Laponite®, both at pH 7.4 and with 10 mM NaCl.

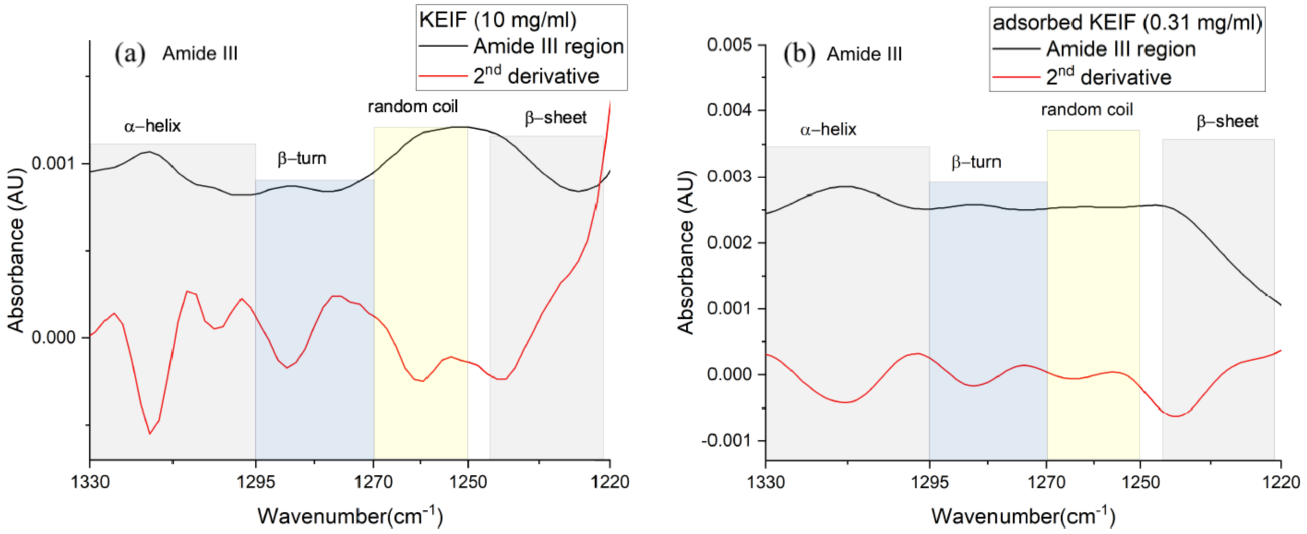

Supplementary Figure 4. Amide III region (black line) of KEIF in-solution 10 mg/ml (a) and adsorbed 0.31 mg/ml (b) together with corresponding second derivative (red line) showing different structural components. α-helix: 1330 -1295 cm^{-1}; β-turn : 1295 - 1270 cm^{-1}; random coil: 1270-1250 cm^{-1}; β-sheet: 1245 - 1220 cm^{-1} (Singh, 1999).

3 MD simulations

3.1 Setup of system

The initial linear structure of KEIF was positioned with the backbone in parallel to the surface and mid-way between the surface and its periodic image along the *z*-coordinate. The Laponite® surface was obtained by replicating the unit cell 25 x 15 times. The Laponite® unit cell is based on that for talc, as defined within the CLAYFF framework (Cygan et al., 2004), but with one magnesium exchanged to a lithium. The charges of the neighbouring oxygen to the substitution site were modified to obtain a net charge of -1 for the unit cell. Thus, the resulting surface charge density was -0.021e/$Å^3$. The composition of the unit cell with the charge and mass of each atom is specified in Figure S5, and the position of each atom is reported in Figure S6.

3.2 Simulation analysis

The variation of the radius of gyration with time for each simulation and its replicates are presented in Figure S7-13. The distribution of the end-to-end distance, radius of gyration and persistence length for each simulation and its replicates are seen in Figure S14-27. Based on these plots, replicates with narrow distributions were consider deviant as the peptide became stuck in one conformation, which would then become overrepresented if considered in subsequent analysis, and were thus removed. The removed replicas are: for KEIF in solution with simulation A (replica 6), E (replica 4), and G (replica 4), and for KEIF adsorbed to Laponite® with simulation C (replica 3) and E (replica 1). A concatenated trajectory was done for each simulation disregarding the before mentioned replicas. All analysis considering averages were done for those trajectories.

The convergence of the simulations was estimated by calculation of the autocorrelation and error estimate from block averaging of the end-to-end distance and radius of gyration, seen in Figures S28-41.

The average Ramachandran plots and their integrated values, comparing that of KEIF in solution with KEIF adsorbed, are seen in Figure S42 and S43. Integration of Ramachandran plots were done in the following four identified regions: I) φ=[-180°,-100°], ψ=[90°,180°], II) φ=[-100,-30], ψ=[90°,180°], III) φ=[-160°,-30°], ψ=[-60°,30°], and IV) φ=[30°,90°], ψ=[-30°,75°].

In Figure S44-57 we present the distribution of secondary structures along the peptide sequence, as determined by DSSPPII analysis, for each simulation and their replicates as well their average. The average DSSPPII structure across the peptide sequence are seen in Figure S58.

Figure S59 shows the average distance from the Laponite surface for each residue and its standard deviation, calculated from the change in minimum distance between each pair over time for the force field/water models A, C, D, and F. Figure S60 shows the probability for each residue of KEIF to hydrogen bond to the Laponite® surface, and Figure S61 the probability for multiple hydrogen bonds between the two, for the force field/water models A, C, D, and F.

```
;Laponite unitcell itp-file

[ moleculetype ]
; molname   nrexcl
LAP       1

[ atoms ]
; id    attype  resnr resname  atname  cgnr  charge       mass
1        mgo    1     LAP      MGO      1    1.3600    24.30500
2        lio    2     LAP      LIO      2    0.5250    24.30500
3        mgo    3     LAP      MGO      3    1.3600    24.30500
4        mgo    4     LAP      MGO      4    1.3600    24.30500
5        mgo    5     LAP      MGO      5    1.3600    24.30500
6        mgo    6     LAP      MGO      6    1.3600    24.30500
7         st    7     LAP       ST      7    2.1000    28.08550
8         st    8     LAP       ST      8    2.1000    28.08550
9         st    9     LAP       ST      9    2.1000    28.08550
10        st   10     LAP       ST     10    2.1000    28.08550
11        st   11     LAP       ST     11    2.1000    28.08550
12        st   12     LAP       ST     12    2.1000    28.08550
13        st   13     LAP       ST     13    2.1000    28.08550
14        st   14     LAP       ST     14    2.1000    28.08550
15      obss   15     LAP     OBSS     15   -1.2996    15.99940
16      obss   16     LAP     OBSS     16   -1.2996    15.99940
17     obsss   17     LAP    OBSSS     17   -1.3043    15.99940
18     obsss   18     LAP    OBSSS     18   -1.3043    15.99940
19     obsss   19     LAP    OBSSS     19   -1.3043    15.99940
20     obsss   20     LAP    OBSSS     20   -1.3043    15.99940
21      obss   21     LAP     OBSS     21   -1.2996    15.99940
22      obss   22     LAP     OBSS     22   -1.2996    15.99940
23  ob_clay    23     LAP  OB_CLAY     23   -1.0500    15.99940
24  ob_clay    24     LAP  OB_CLAY     24   -1.0500    15.99940
25  ob_clay    25     LAP  OB_CLAY     25   -1.0500    15.99940
26  ob_clay    26     LAP  OB_CLAY     26   -1.0500    15.99940
27  ob_clay    27     LAP  OB_CLAY     27   -1.0500    15.99940
28  ob_clay    28     LAP  OB_CLAY     28   -1.0500    15.99940
29  ob_clay    29     LAP  OB_CLAY     29   -1.0500    15.99940
30  ob_clay    30     LAP  OB_CLAY     30   -1.0500    15.99940
31  ob_clay    31     LAP  OB_CLAY     31   -1.0500    15.99940
32  ob_clay    32     LAP  OB_CLAY     32   -1.0500    15.99940
33  ob_clay    33     LAP  OB_CLAY     33   -1.0500    15.99940
34  ob_clay    34     LAP  OB_CLAY     34   -1.0500    15.99940
35        oh   35     LAP      OHS     35   -0.9547    15.99940
36        oh   36     LAP       OH     36   -0.9500    15.99940
37        oh   37     LAP       OH     37   -0.9500    15.99940
38        oh   38     LAP      OHS     38   -0.9547    15.99940
39        ho   39     LAP       HO     39    0.4250    1.007940
40        ho   40     LAP       HO     40    0.4250    1.007940
41        ho   41     LAP       HO     41    0.4250    1.007940
42        ho   42     LAP       HO     42    0.4250    1.007940

[ bonds ]
; i    j    type
35       39     1         0.1    463532.808
36       40     1         0.1    463532.808
37       41     1         0.1    463532.808
38       42     1         0.1    463532.808
```

Supplementary Figure 5. The *itp-file* for the Laponite® unit cell which defines the charges and mass of each of its atoms.

```
Laponite unitcell gro-file
42
    1LAP          MGO    1   0.206   0.227   0.500
    1LAP          LIO    2   0.206   0.530   0.500
    1LAP          MGO    3   0.206   0.834   0.500
    1LAP          MGO    4   0.468   0.076   0.500
    1LAP          MGO    5   0.468   0.379   0.500
    1LAP          MGO    6   0.468   0.682   0.500
    1LAP           ST    7   0.030   0.227   0.228
    1LAP           ST    8   0.030   0.833   0.228
    1LAP           ST    9   0.292   0.379   0.228
    1LAP           ST    10   0.292   0.682   0.228
    1LAP           ST    11   0.119   0.379   0.772
    1LAP           ST    12   0.119   0.682   0.772
    1LAP           ST    13   0.381   0.227   0.772
    1LAP           ST    14   0.381   0.833   0.772
    1LAP         OBSS    15   0.031   0.833   0.388
    1LAP         OBSS    16   0.031   0.227   0.388
    1LAP       OBSSS    17   0.293   0.378   0.388
    1LAP       OBSSS    18   0.293   0.682   0.388
    1LAP       OBSSS    19   0.119   0.378   0.612
    1LAP       OBSSS    20   0.119   0.682   0.612
    1LAP         OBSS    21   0.381   0.227   0.612
    1LAP         OBSS    22   0.381   0.833   0.612
    1LAP     OB_CLAY    23   0.162   0.301   0.168
    1LAP     OB_CLAY    24   0.162   0.759   0.168
    1LAP     OB_CLAY    25   0.290   0.530   0.168
    1LAP     OB_CLAY    26   0.424   0.304   0.168
    1LAP     OB_CLAY    27   0.424   0.756   0.168
    1LAP     OB_CLAY    28   0.552   0.076   0.168
    1LAP     OB_CLAY    29   0.122   0.530   0.832
    1LAP     OB_CLAY    30   0.249   0.301   0.832
    1LAP     OB_CLAY    31   0.249   0.759   0.832
    1LAP     OB_CLAY    32   0.384   0.076   0.832
    1LAP     OB_CLAY    33   0.511   0.756   0.832
    1LAP     OB_CLAY    34   0.511   0.304   0.832
    1LAP          OHS    35   0.032   0.530   0.400
    1LAP           OH    36   0.294   0.076   0.400
    1LAP           OH    37   0.118   0.076   0.600
    1LAP          OHS    38   0.380   0.530   0.600
    1LAP           HO    39   0.032   0.530   0.300
    1LAP           HO    40   0.294   0.076   0.300
    1LAP           HO    41   0.118   0.076   0.700
    1LAP           HO    42   0.380   0.530   0.700
   0.52400   0.90900   1.00000
```

Supplementary Figure 6. The structure file of the Laponite® unit cell used in simulations, with the *x*-, *y*- and *z*-coordinates shown in the last three columns.

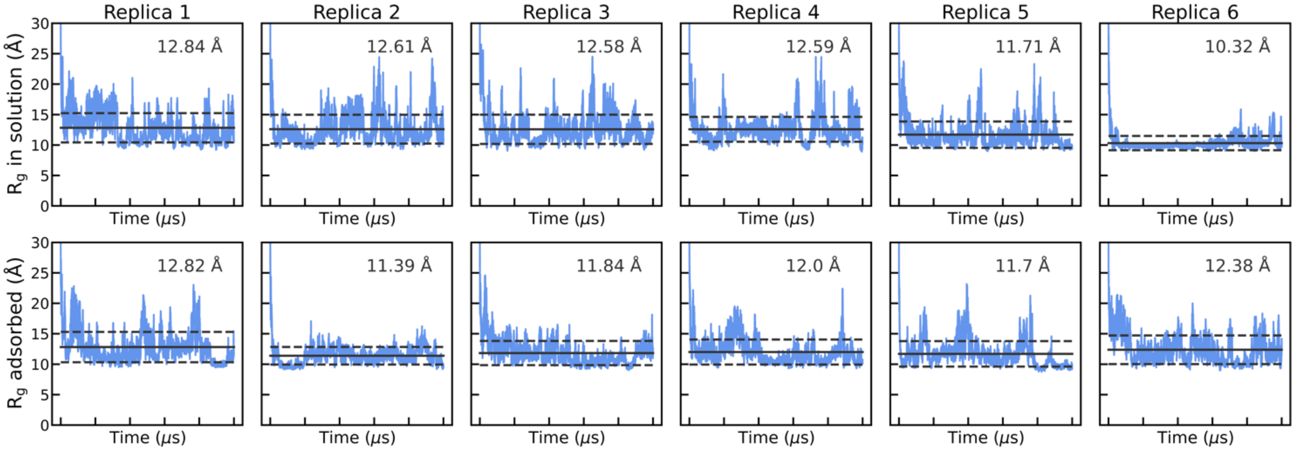

Supplementary Figure 7. The radius of gyration, R_g, over time for KEIF in solution (top) and adsorbed (bottom) for all six replicas of simulation A.

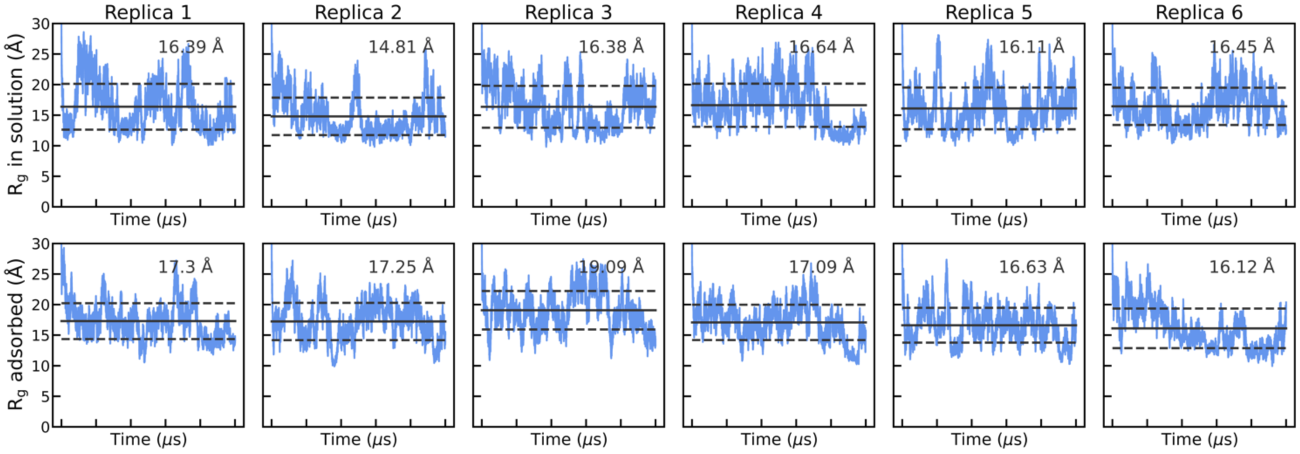

Supplementary Figure 8. The radius of gyration, R_g, over time for KEIF in solution (top) and adsorbed (bottom) for all six replicas of simulation B.

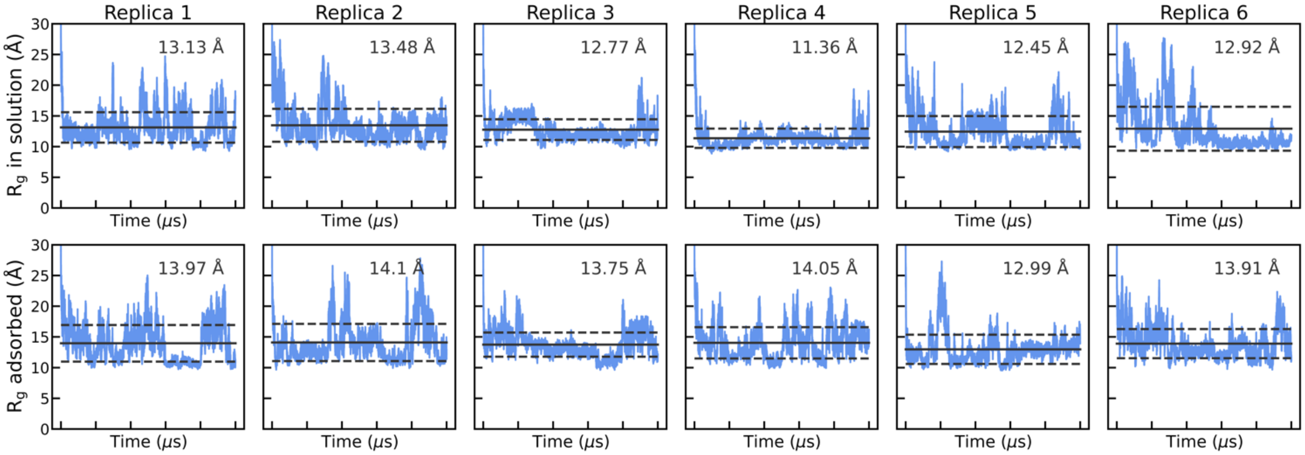

Supplementary Figure 9. The radius of gyration, R_g, over time for KEIF in solution (top) and adsorbed (bottom) for all six replicas of simulation C.

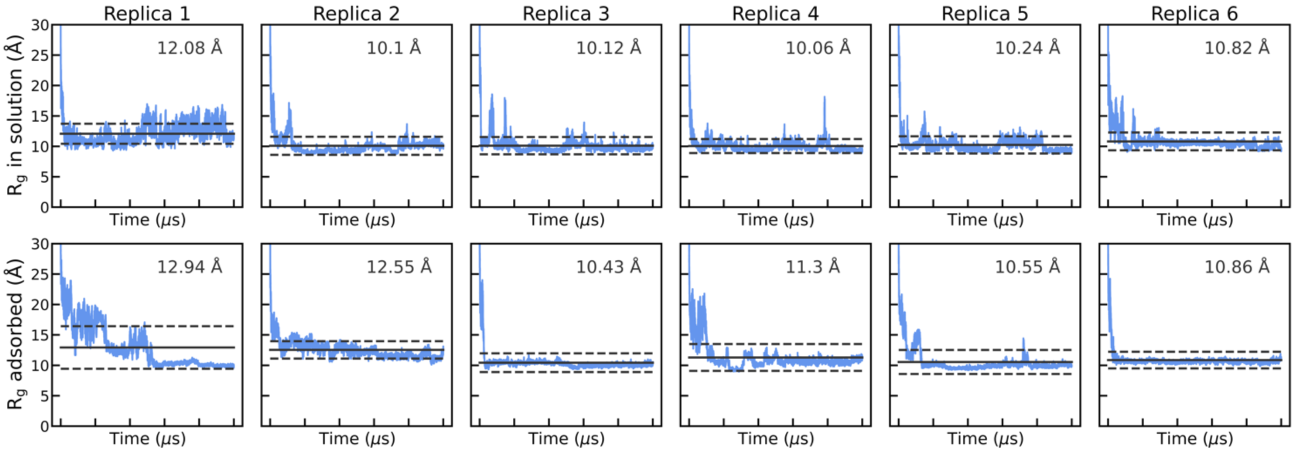

Supplementary Figure 10. The radius of gyration, R_g, over time for KEIF in solution (top) and adsorbed (bottom) for all six replicas of simulation D.

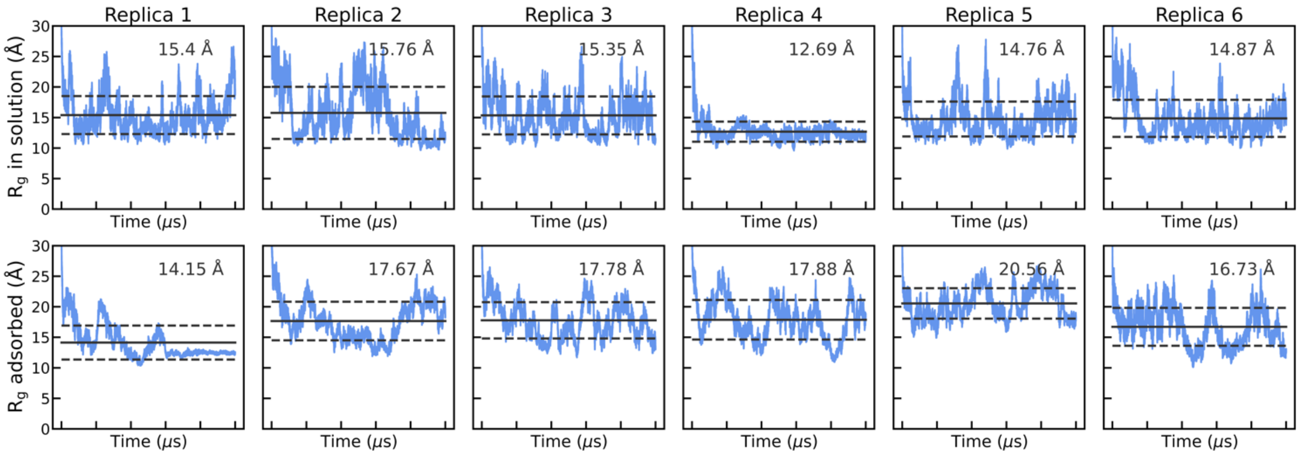

Supplementary Figure 11. The radius of gyration, R_g, over time for KEIF in solution (top) and adsorbed (bottom) for all six replicas of simulation E.

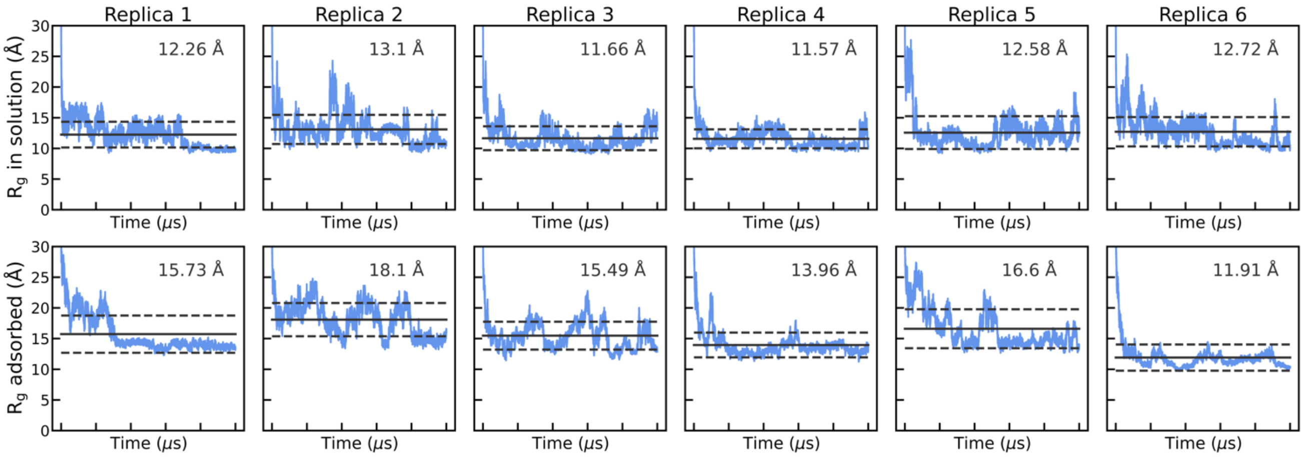

Supplementary Figure 12. The radius of gyration, R_g, over time for KEIF in solution (top) and adsorbed (bottom) for all six replicas of simulation F.

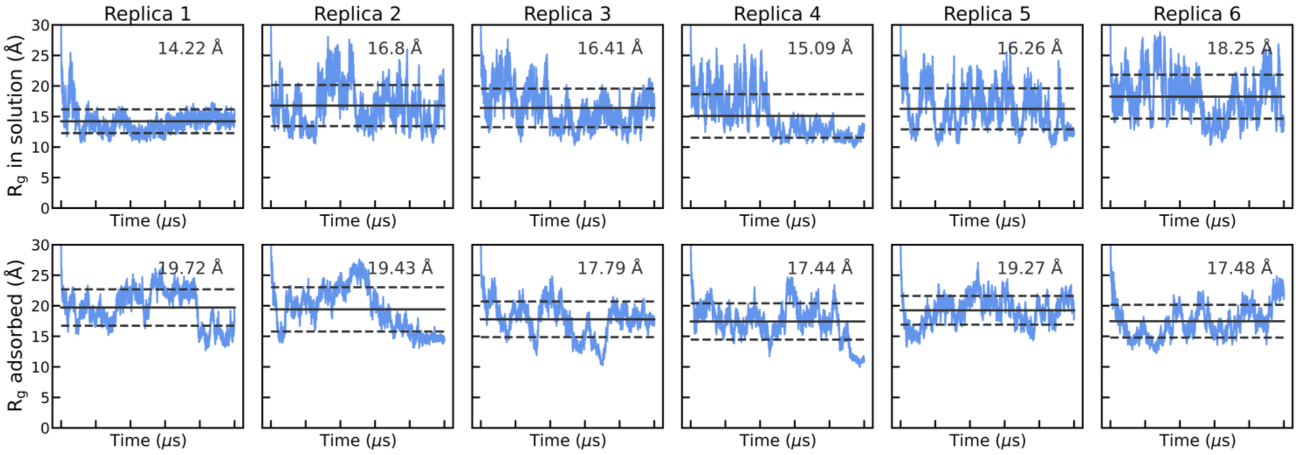

Supplementary Figure 13. The radius of gyration, R_g, over time for KEIF in solution (top) and adsorbed (bottom) for all six replicas of simulation G.

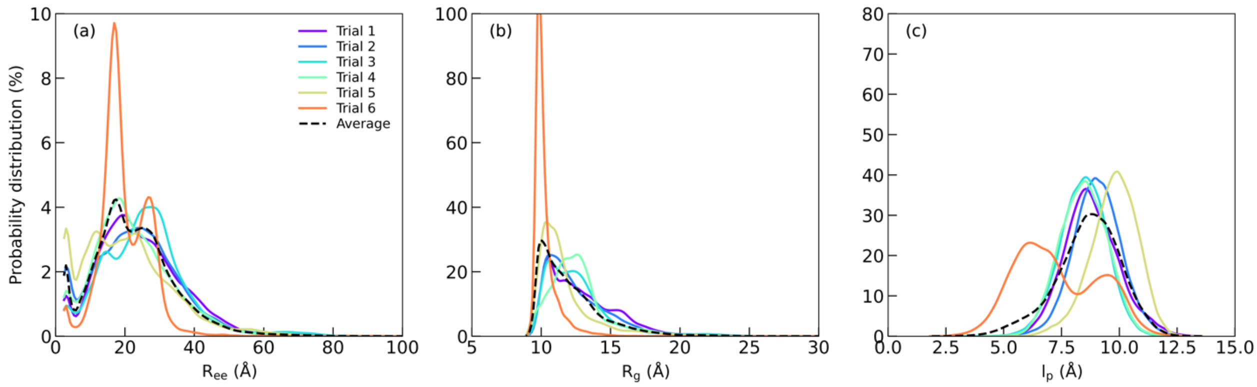

Supplementary Figure 14. The distribution of a) the end-to-end distance, R_{ee}, b) the radius of gyration, R_g, and c) the persistence length, l_p, for each replicate of KEIF in solution with the force field/water model A.

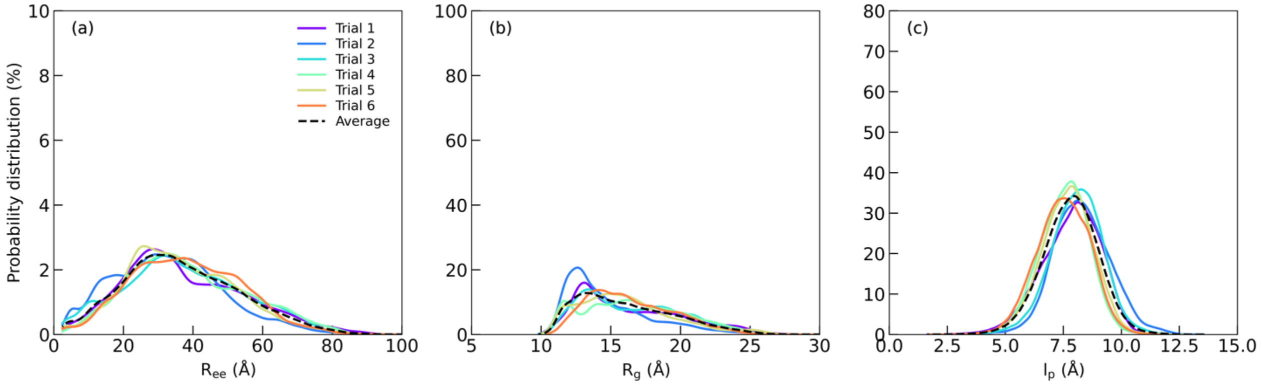

Supplementary Figure 15. The distribution of a) the end-to-end distance, R_{ee}, b) the radius of gyration, R_g, and c) the persistence length, l_p, for each replicate of KEIF in solution with the force field/water model B.

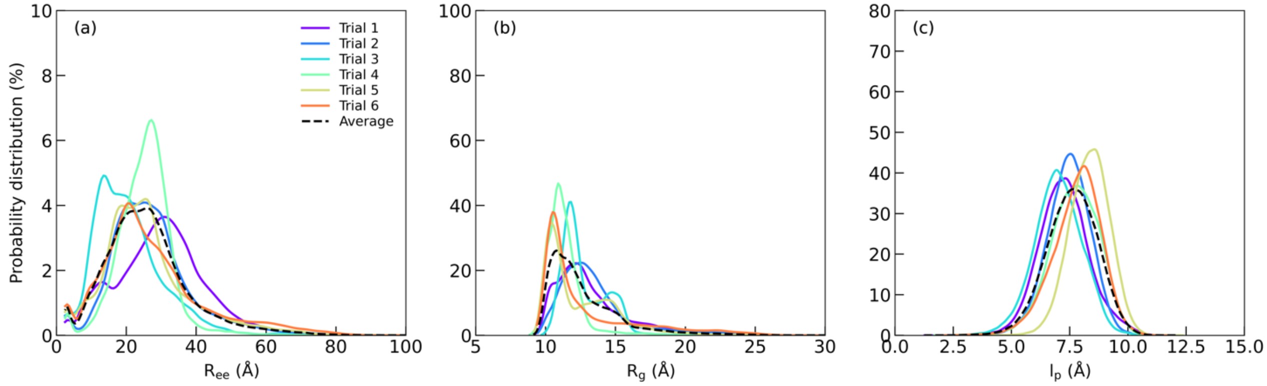

Supplementary Figure 16. The distribution of a) the end-to-end distance, R_{ee}, b) the radius of gyration, R_g, and c) the persistence length, l_p, for each replicate of KEIF in solution with the force field/water model C.

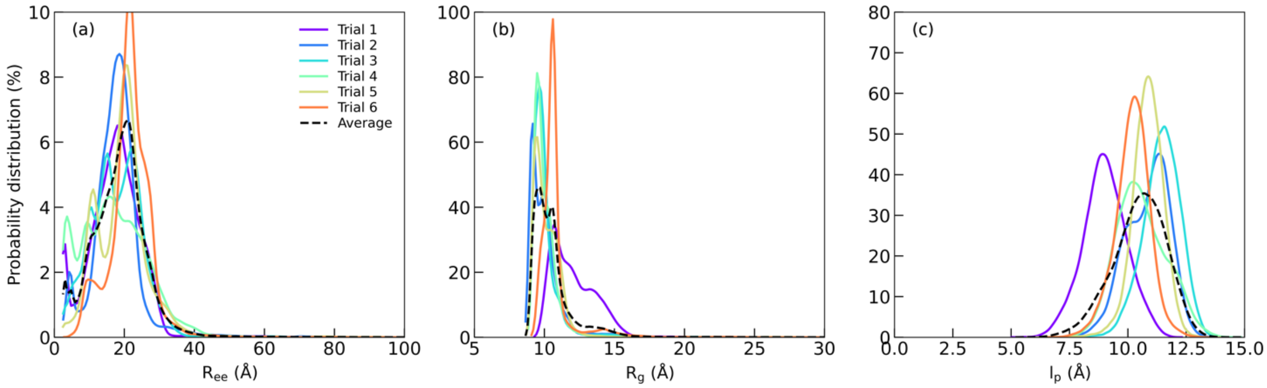

Supplementary Figure 17. The distribution of a) the end-to-end distance, R_{ee}, b) the radius of gyration, R_g, and c) the persistence length, l_p, for each replicate of KEIF in solution with the force field/water model D.

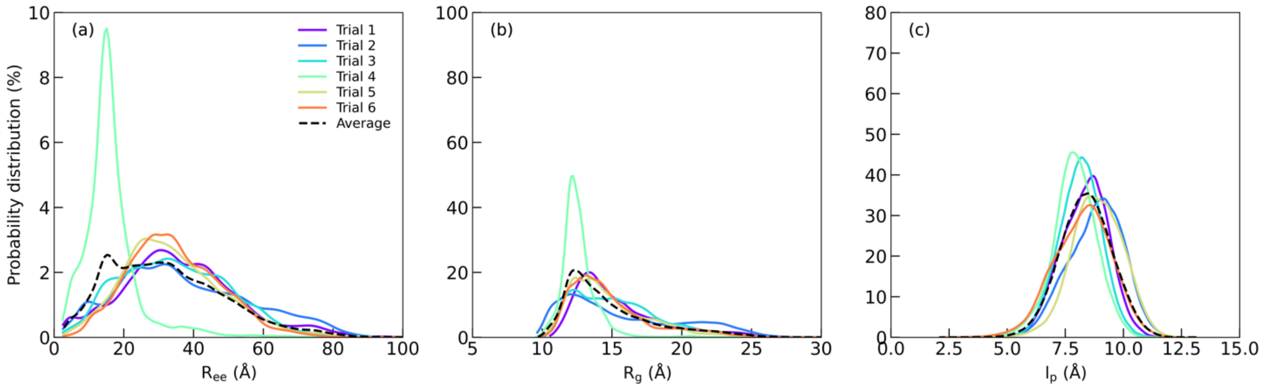

Supplementary Figure 18. The distribution of a) the end-to-end distance, R_{ee}, b) the radius of gyration, R_g, and c) the persistence length, l_p, for each replicate of KEIF in solution with the force field/water model E.

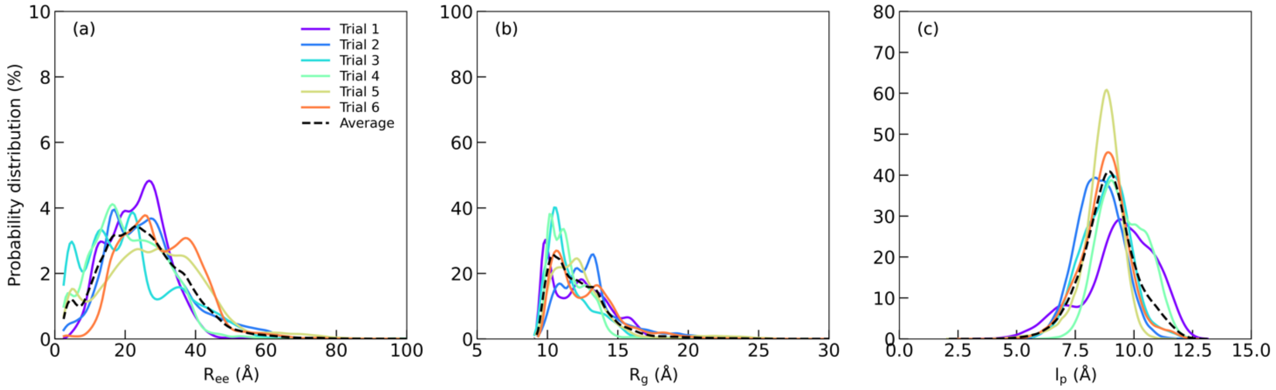

Supplementary Figure 19. The distribution of a) the end-to-end distance, R_{ee}, b) the radius of gyration, R_g, and c) the persistence length, l_p, for each replicate of KEIF in solution with the force field/water model F.

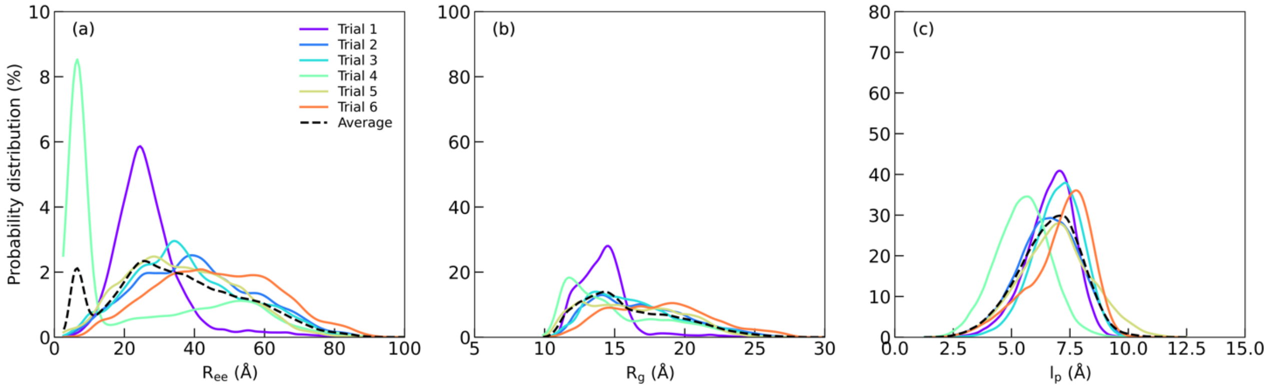

Supplementary Figure 20. The distribution of a) the end-to-end distance, R_{ee}, b) the radius of gyration, R_g, and c) the persistence length, l_p, for each replicate of KEIF in solution with the force field/water model G.

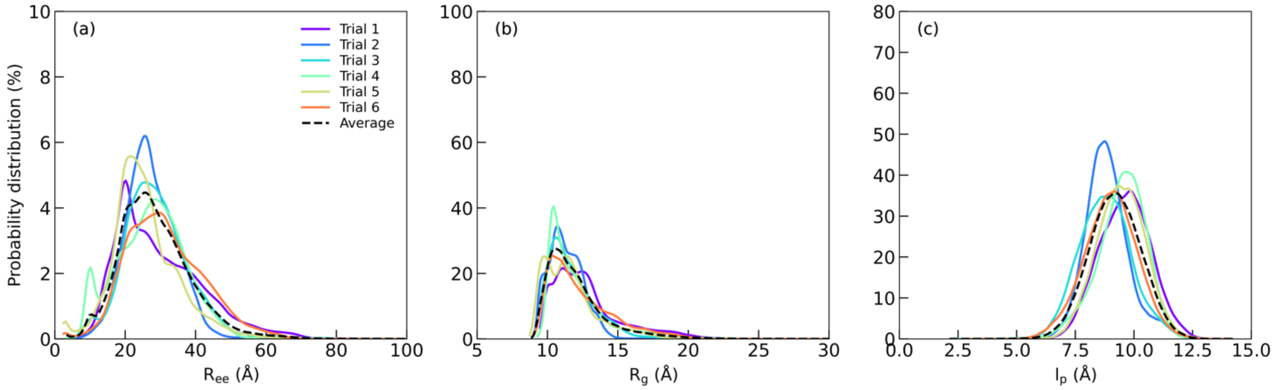

Supplementary Figure 21. The distribution of a) the end-to-end distance, R_{ee}, b) the radius of gyration, R_g, and c) the persistence length, l_p, for each replicate of KEIF adsorbed to Laponite® with the force field/water model A.

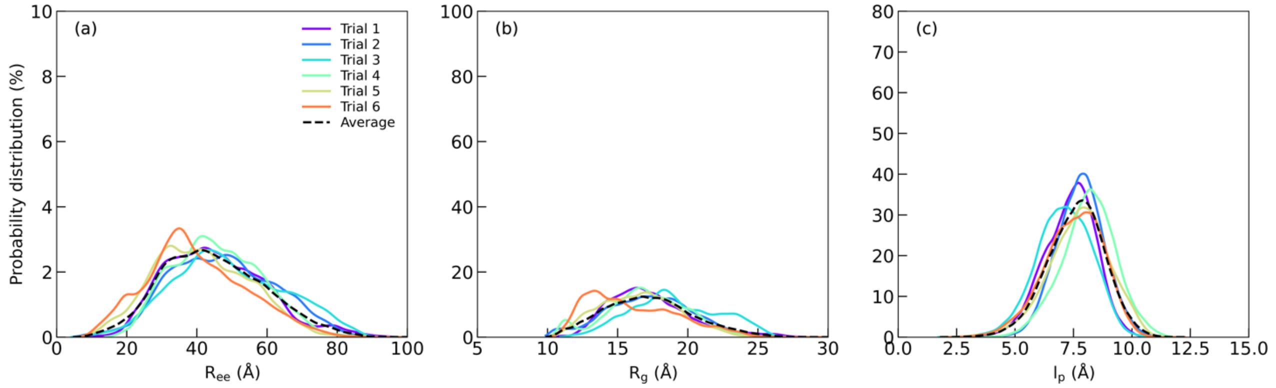

Supplementary Figure 22. The distribution of a) the end-to-end distance, R_{ee}, b) the radius of gyration, R_g, and c) the persistence length, l_p, for each replicate of KEIF adsorbed to Laponite® with the force field/water model B.

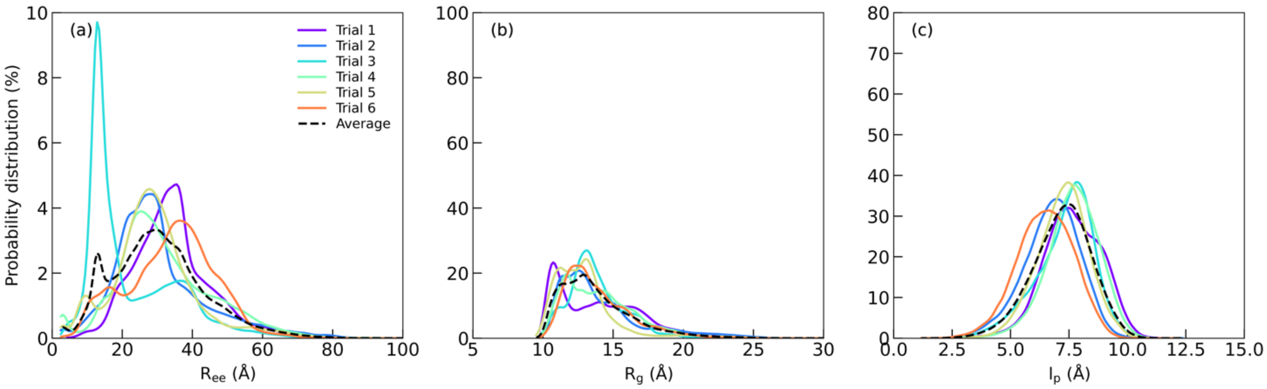

Supplementary Figure 23. The distribution of a) the end-to-end distance, R_{ee}, b) the radius of gyration, R_g, and c) the persistence length, l_p, for each replicate of KEIF adsorbed to Laponite® with the force field/water model C.

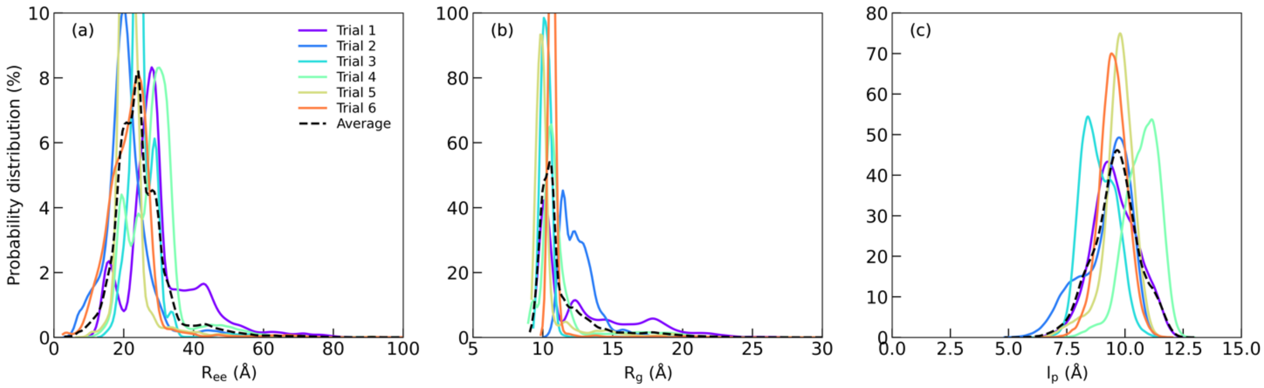

Supplementary Figure 24. The distribution of a) the end-to-end distance, R_{ee}, b) the radius of gyration, R_g, and c) the persistence length, l_p, for each replicate of KEIF adsorbed to Laponite® with the force field/water model D.

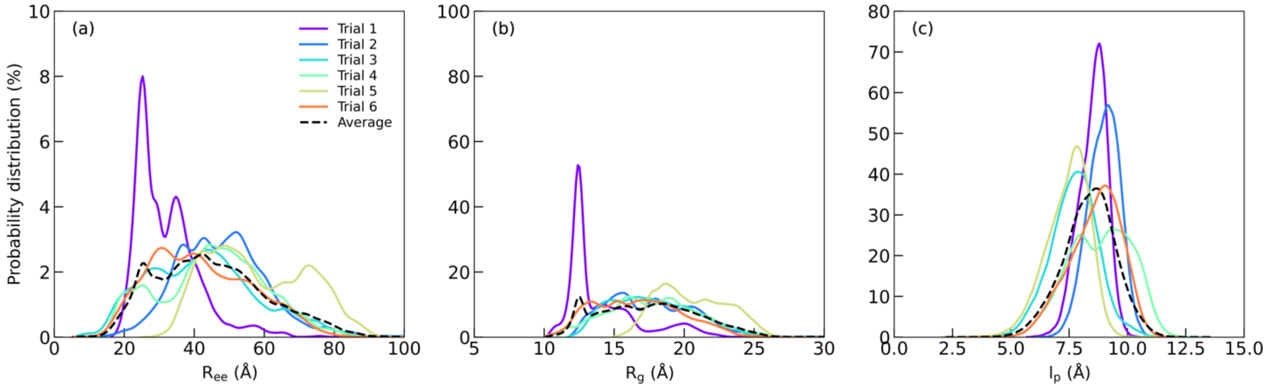

Supplementary Figure 25. The distribution of a) the end-to-end distance, R_{ee}, b) the radius of gyration, R_g, and c) the persistence length, l_p, for each replicate of KEIF adsorbed to Laponite® with the force field/water model E.

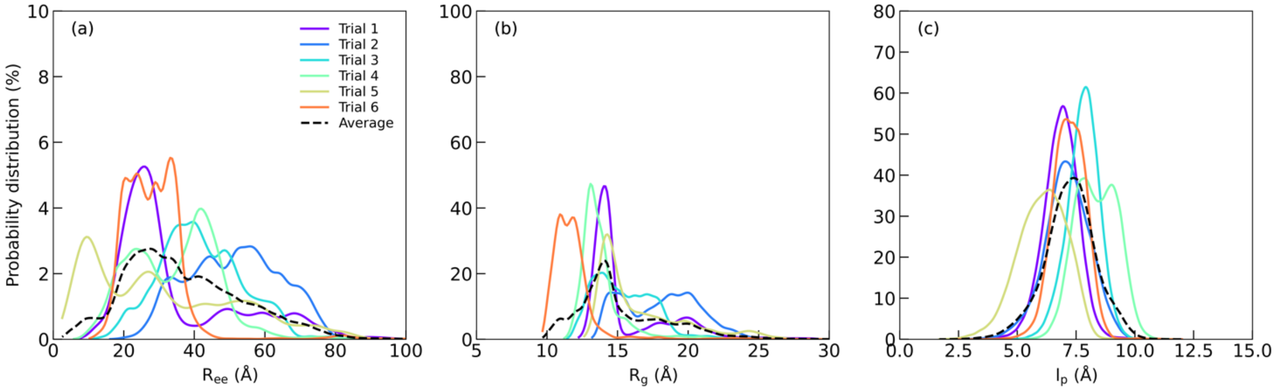

Supplementary Figure 26. The distribution of a) the end-to-end distance, R_{ee}, b) the radius of gyration, R_g, and c) the persistence length, l_p, for each replicate of KEIF adsorbed to Laponite® with the force field/water model F.

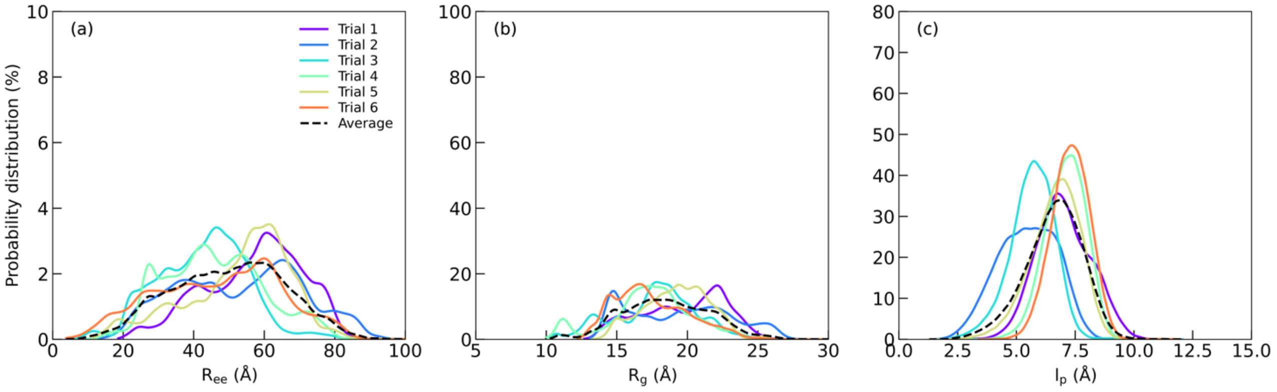

Supplementary Figure 27. The distribution of a) the end-to-end distance, R_{ee}, b) the radius of gyration, R_g, and c) the persistence length, l_p, for each replicate of KEIF adsorbed to Laponite® with the force field/water model G.

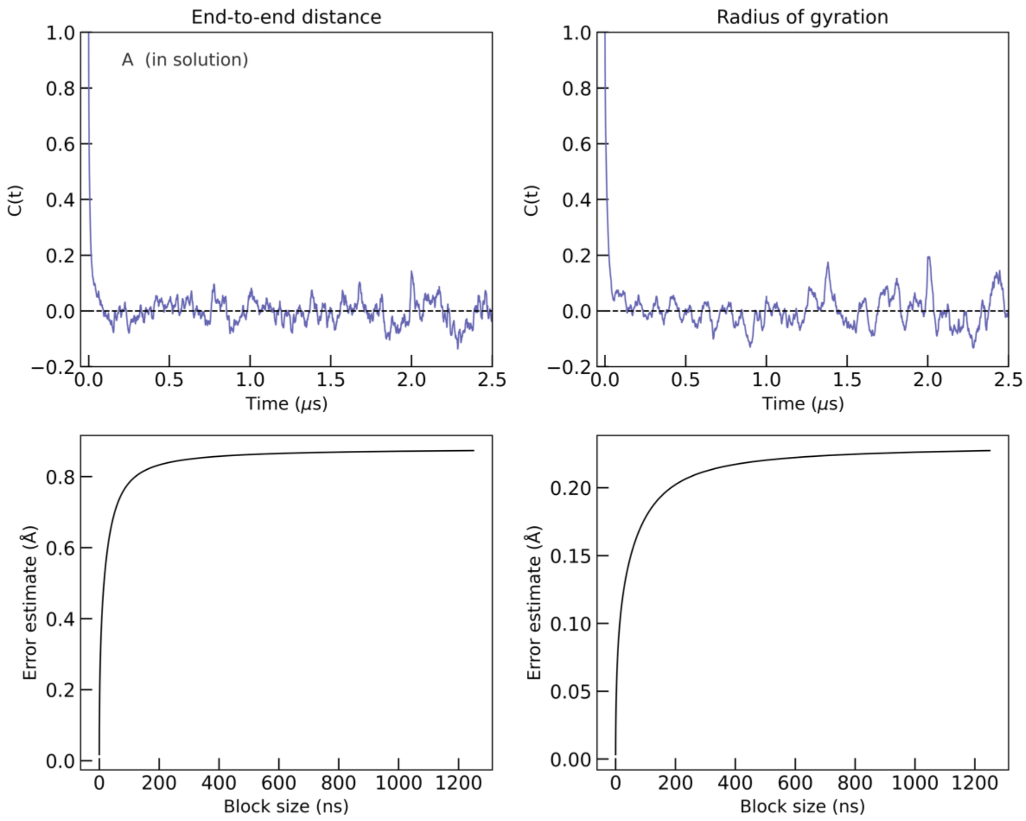

Supplementary Figure 28. Autocorrelation (top) and error estimates (bottom) from block averaging of the end-to-end distance (left) and radius of gyration (right) for the concatenated trajectory of KEIF in solution with the force field/water model A.

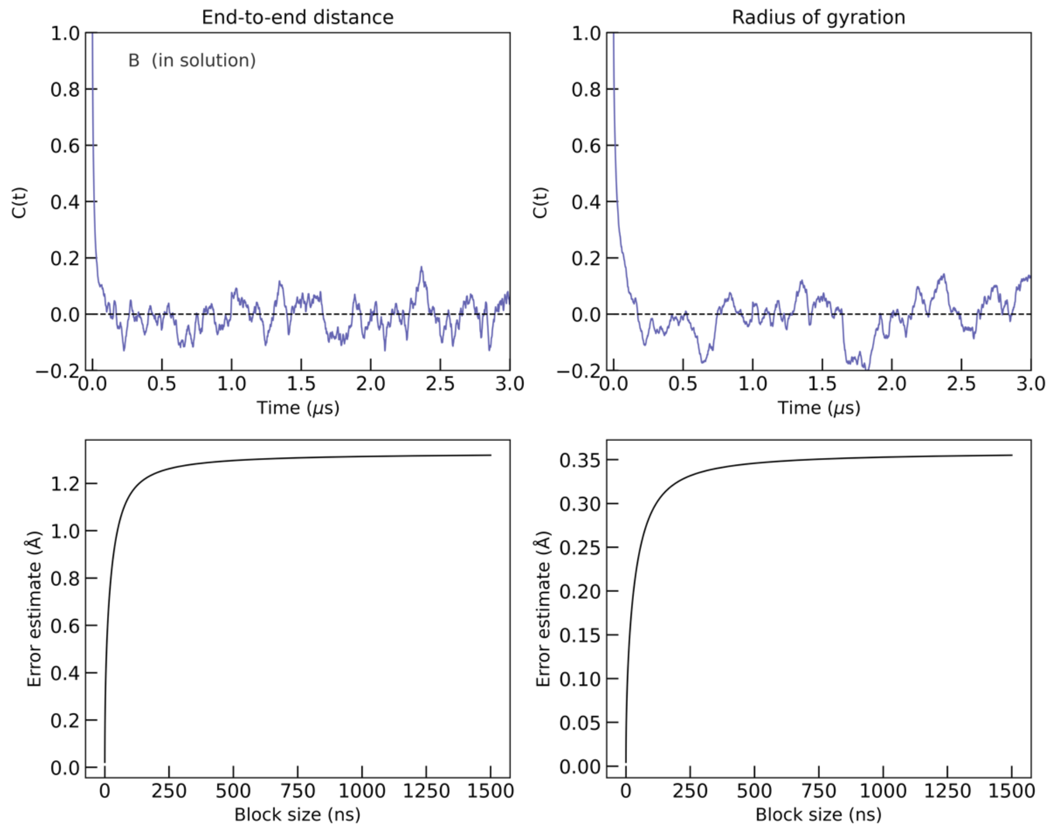

Supplementary Figure 29. Autocorrelation (top) and error estimates (bottom) from block averaging of the end-to-end distance (left) and radius of gyration (right) for the concatenated trajectory of KEIF in solution with the force field/water model B.

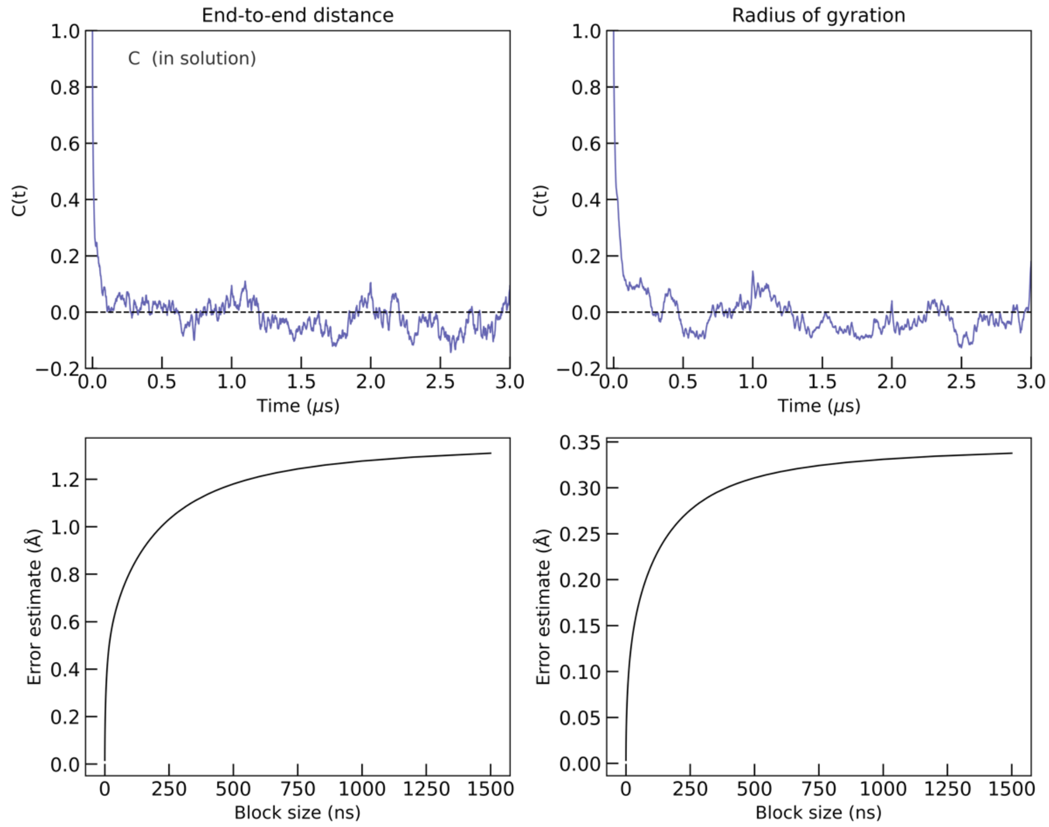

Supplementary Figure 30. Autocorrelation (top) and error estimates (bottom) from block averaging of the end-to-end distance (left) and radius of gyration (right) for the concatenated trajectory of KEIF in solution with the force field/water model C.

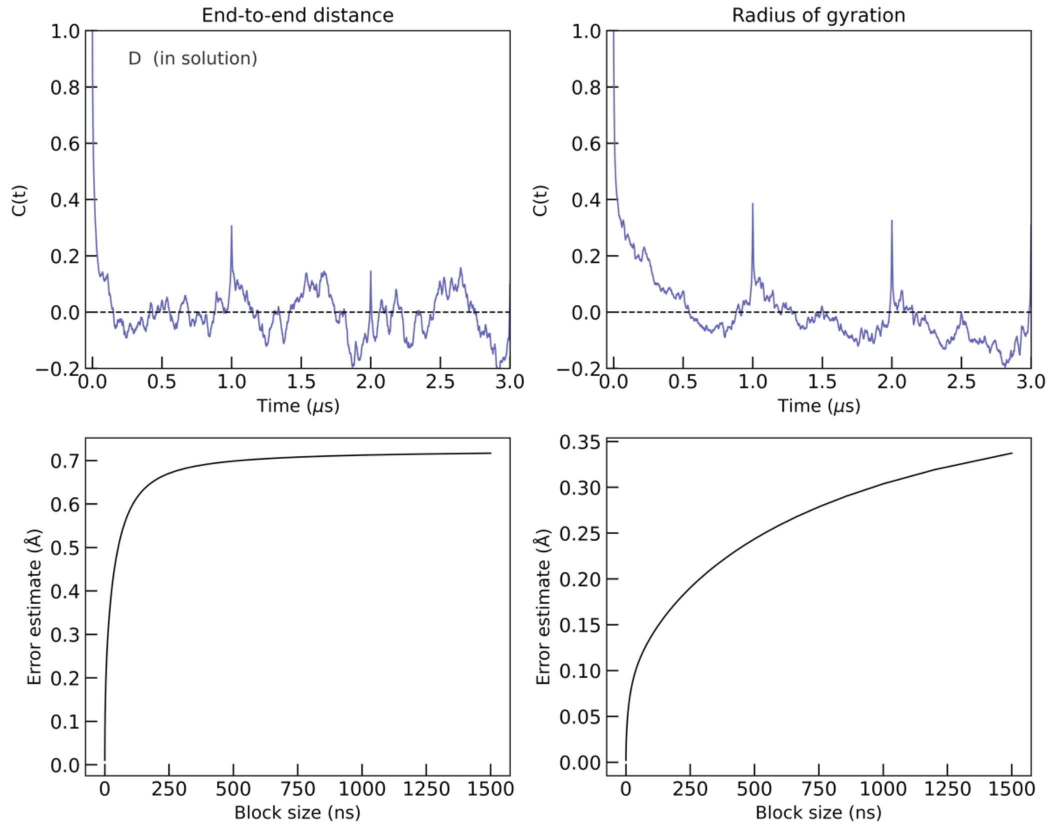

Supplementary Figure 31. Autocorrelation (top) and error estimates (bottom) from block averaging of the end-to-end distance (left) and radius of gyration (right) for the concatenated trajectory of KEIF in solution with the force field/water model D.

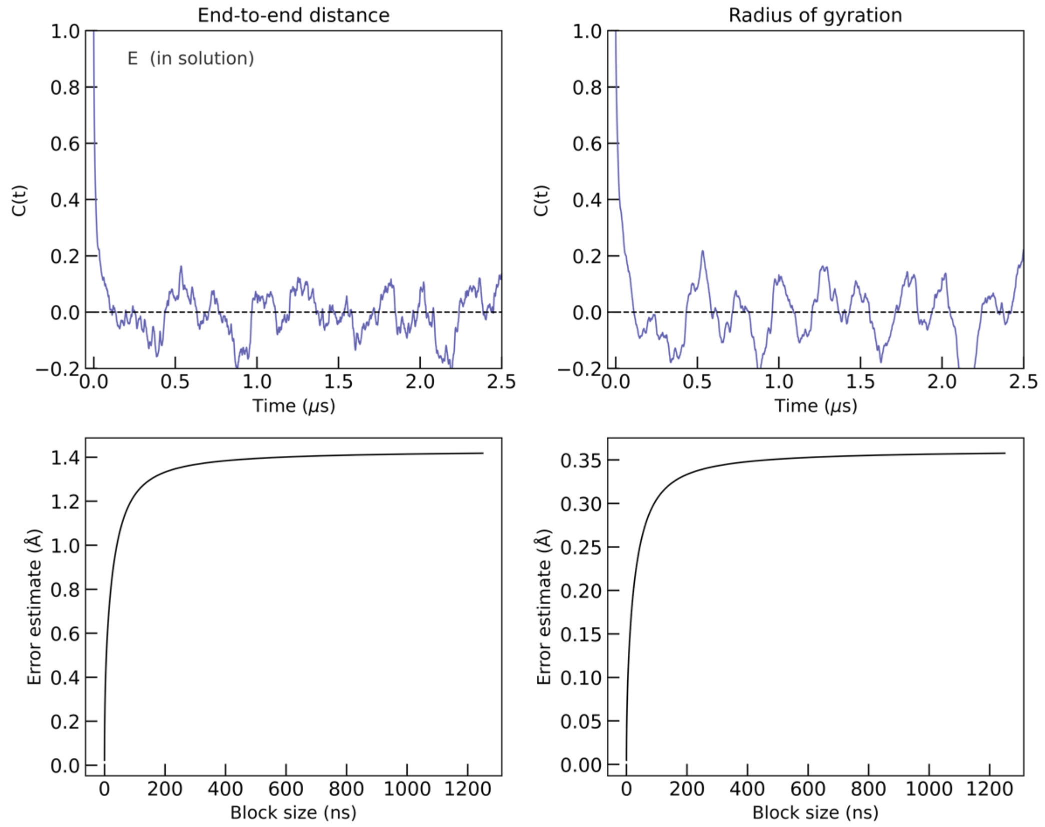

Supplementary Figure 32. Autocorrelation (top) and error estimates (bottom) from block averaging of the end-to-end distance (left) and radius of gyration (right) for the concatenated trajectory of KEIF in solution with the force field/water model E.

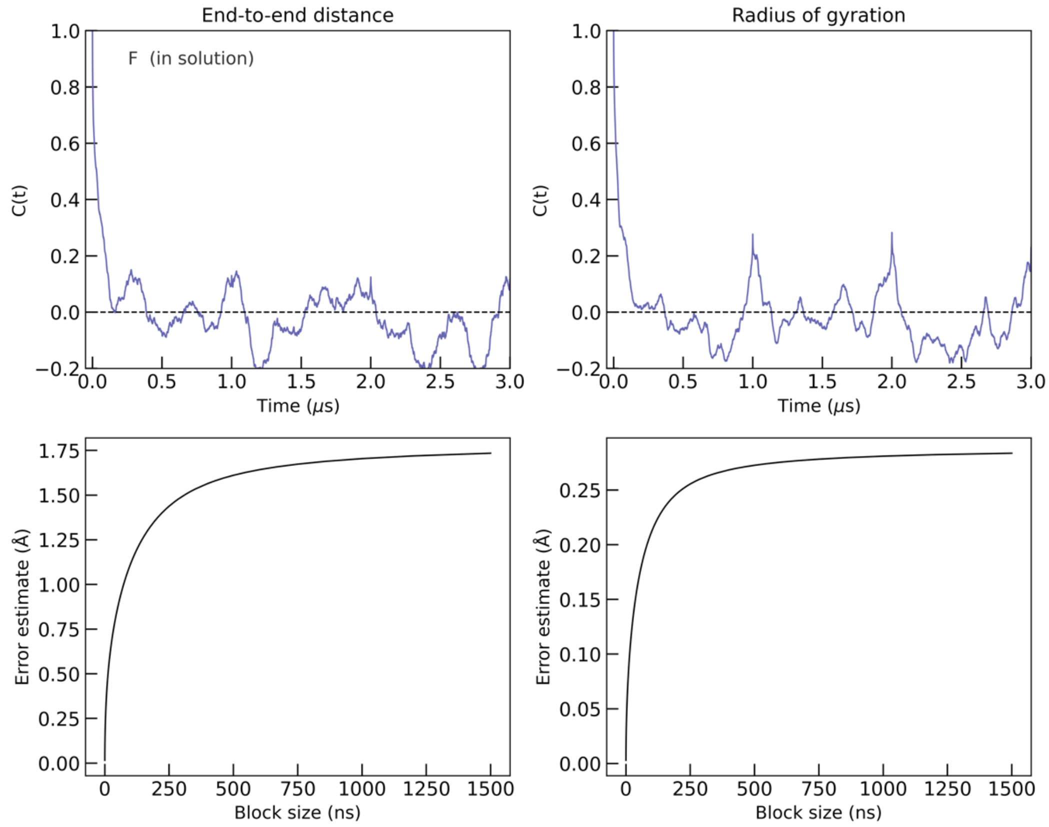

Supplementary Figure 33. Autocorrelation (top) and error estimates (bottom) from block averaging of the end-to-end distance (left) and radius of gyration (right) for the concatenated trajectory of KEIF in solution with the force field/water model F.

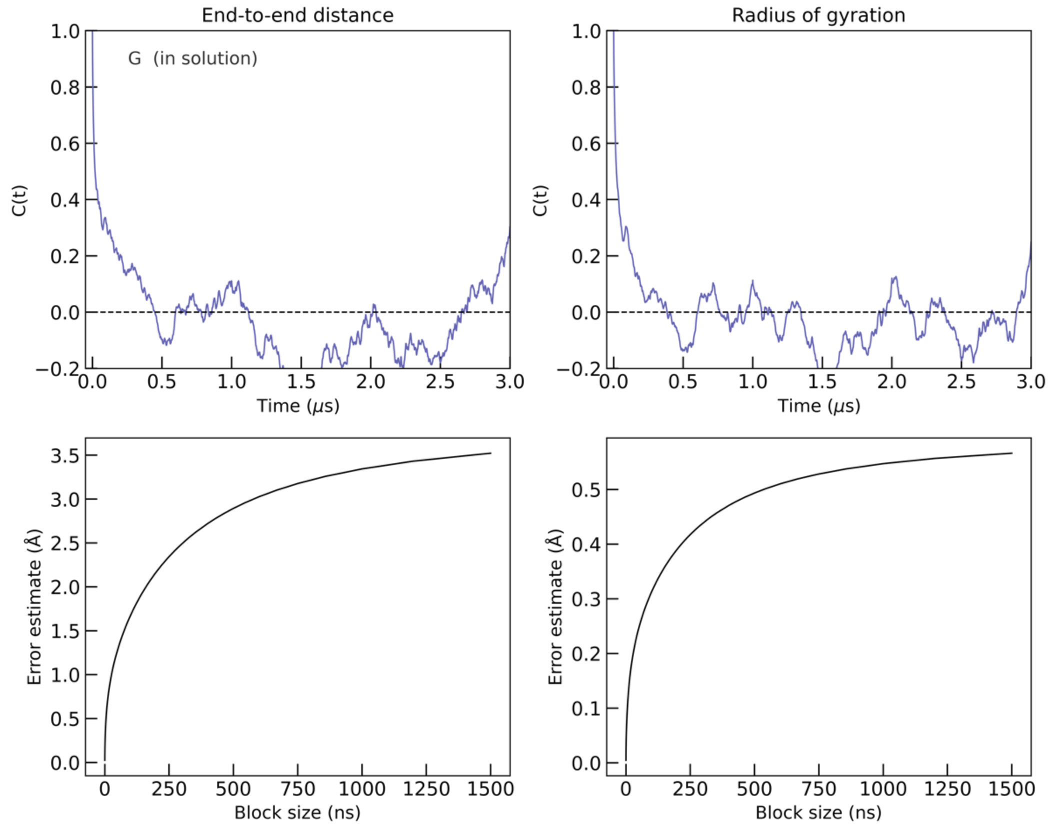

Supplementary Figure 34. Autocorrelation (top) and error estimates (bottom) from block averaging of the end-to-end distance (left) and radius of gyration (right) for the concatenated trajectory of KEIF in solution with the force field/water model G.

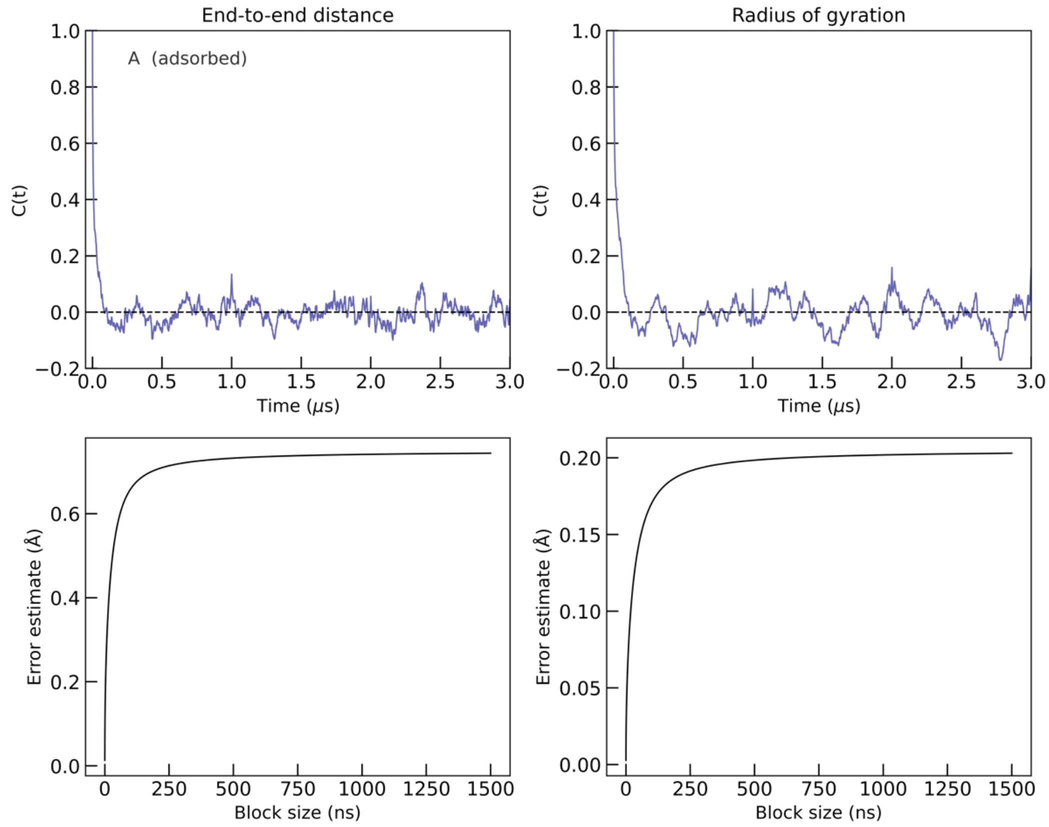

Supplementary Figure 35. Autocorrelation (top) and error estimates (bottom) from block averaging of the end-to-end distance (left) and radius of gyration (right) for the concatenated trajectory of KEIF, adsorbed to Laponite® with the force field/water model A.

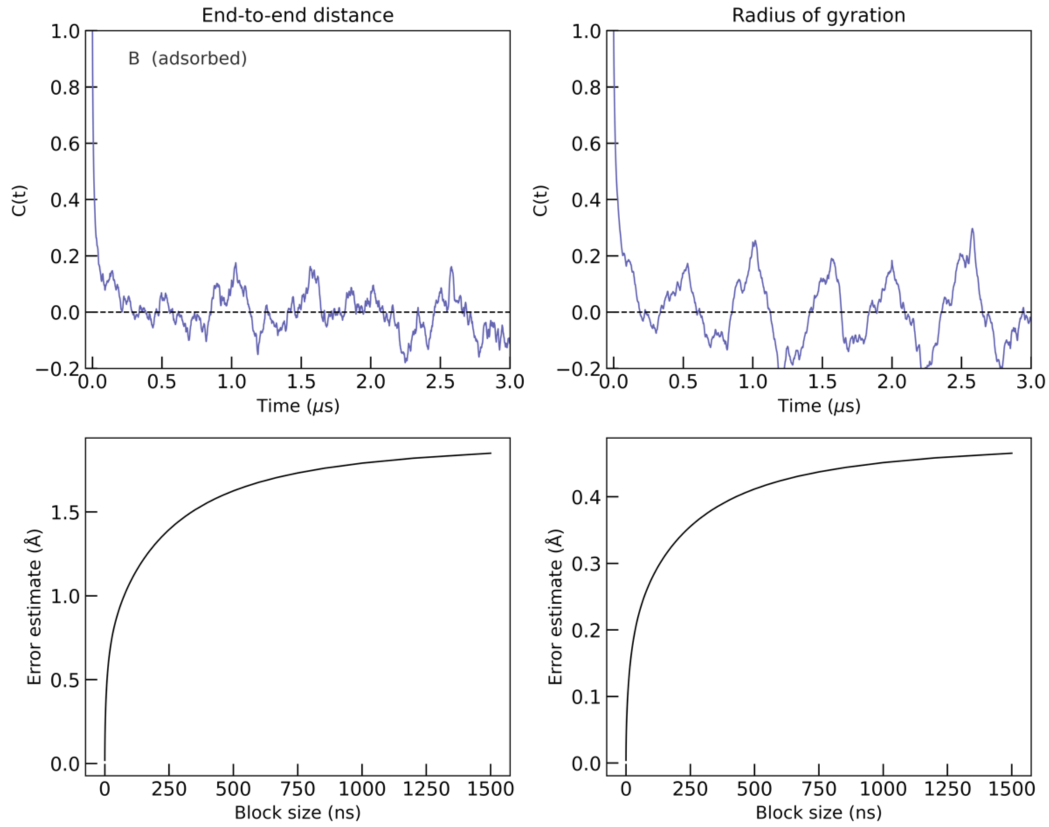

Supplementary Figure 36. Autocorrelation (top) and error estimates (bottom) from block averaging of the end-to-end distance (left) and radius of gyration (right) for the concatenated trajectory of KEIF, adsorbed to Laponite® with the force field/water model B.

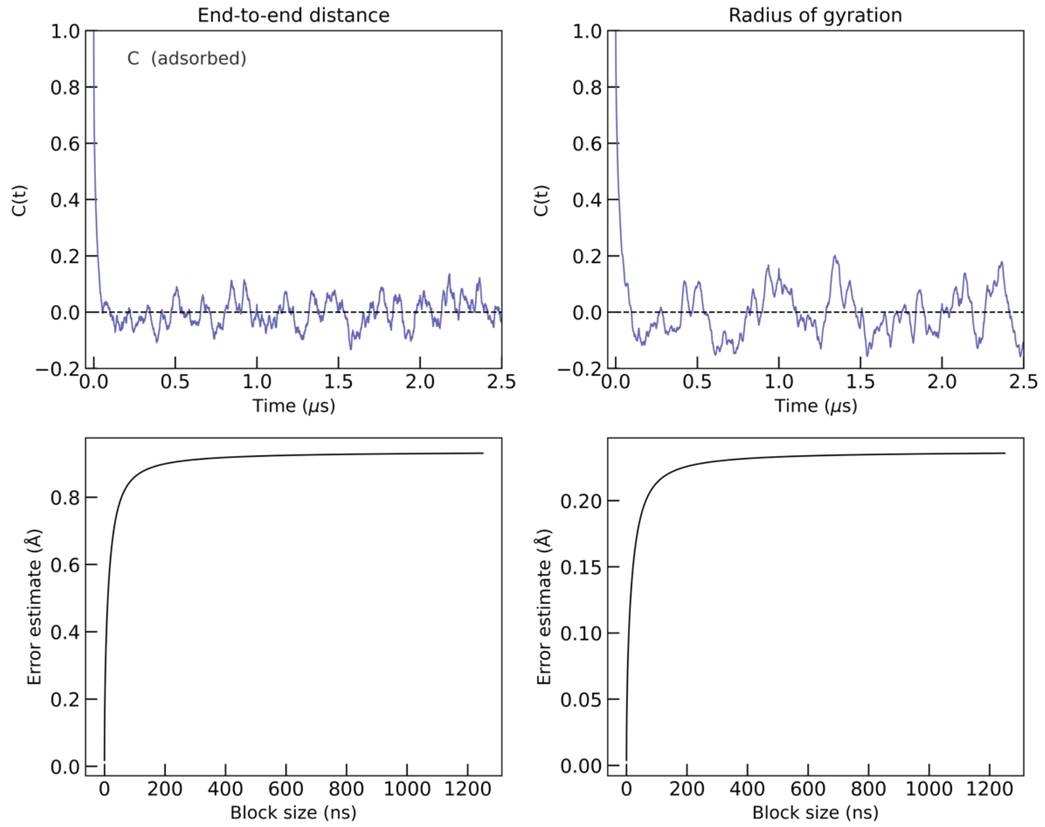

Supplementary Figure 37. Autocorrelation (top) and error estimates (bottom) from block averaging of the end-to-end distance (left) and radius of gyration (right) for the concatenated trajectory of KEIF, adsorbed to Laponite® with the force field/water model C.

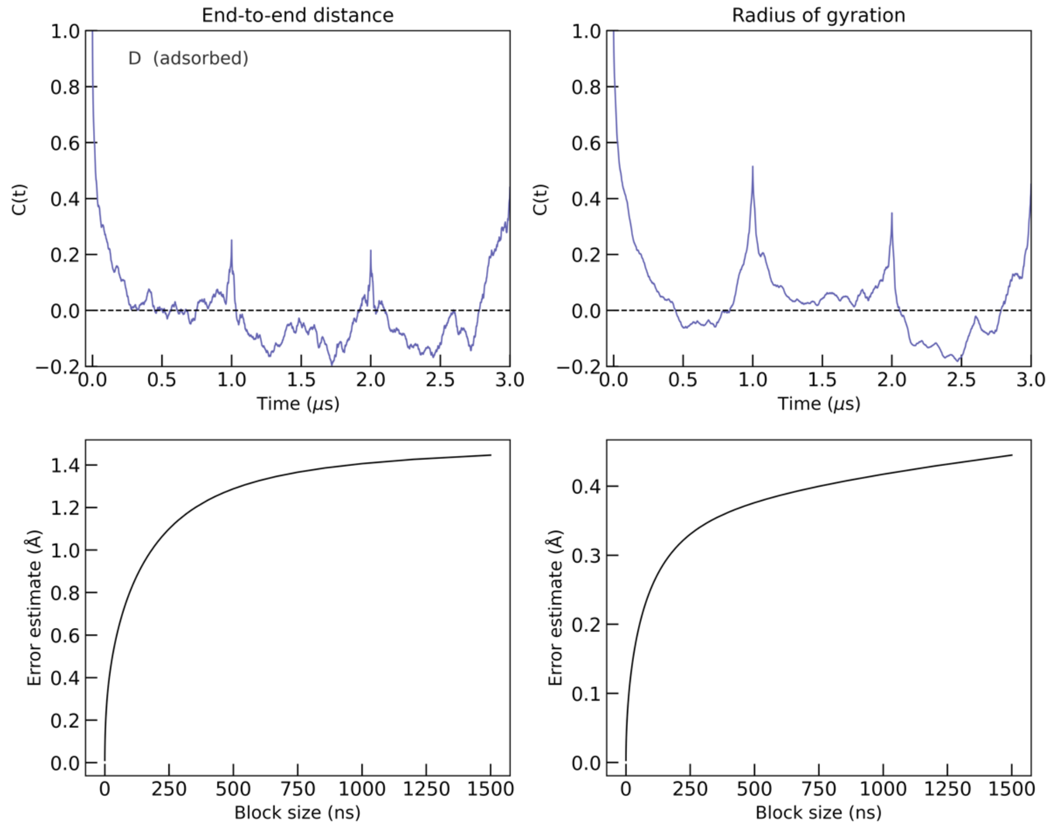

Supplementary Figure 38. Autocorrelation (top) and error estimates (bottom) from block averaging of the end-to-end distance (left) and radius of gyration (right) for the concatenated trajectory of KEIF, adsorbed to Laponite® with the force field/water model D.

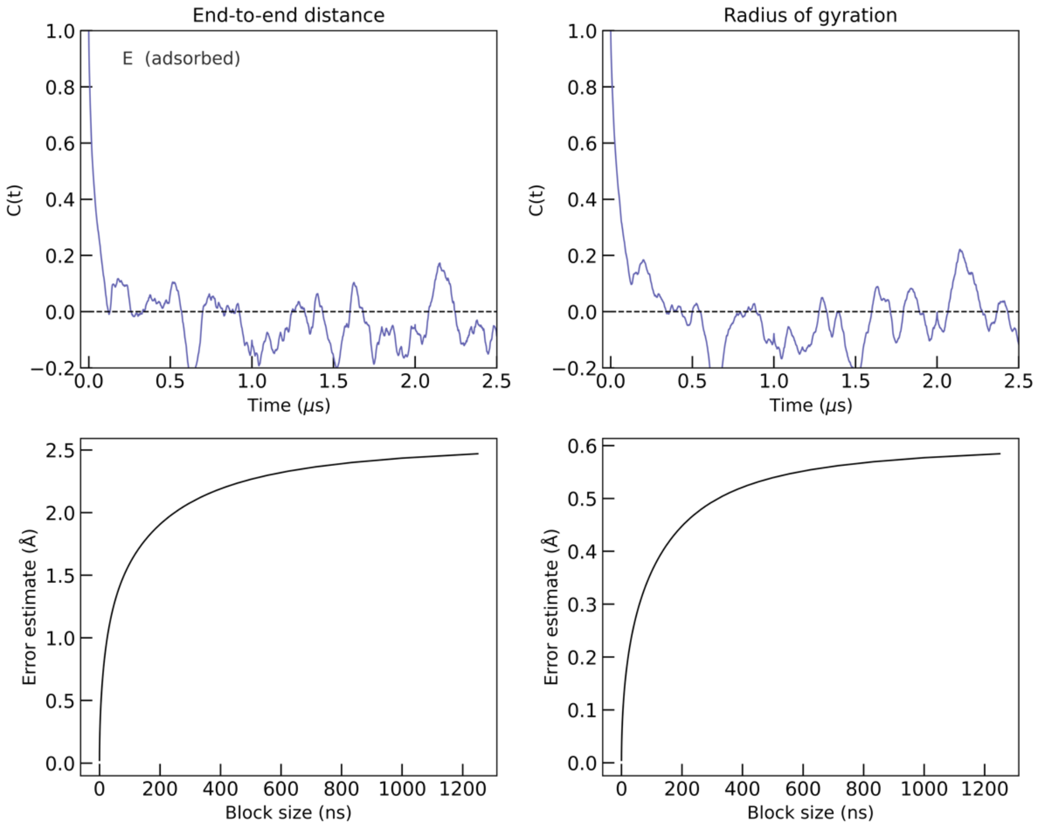

Supplementary Figure 39. Autocorrelation (top) and error estimates (bottom) from block averaging of the end-to-end distance (left) and radius of gyration (right) for the concatenated trajectory of KEIF, adsorbed to Laponite® with the force field/water model E.

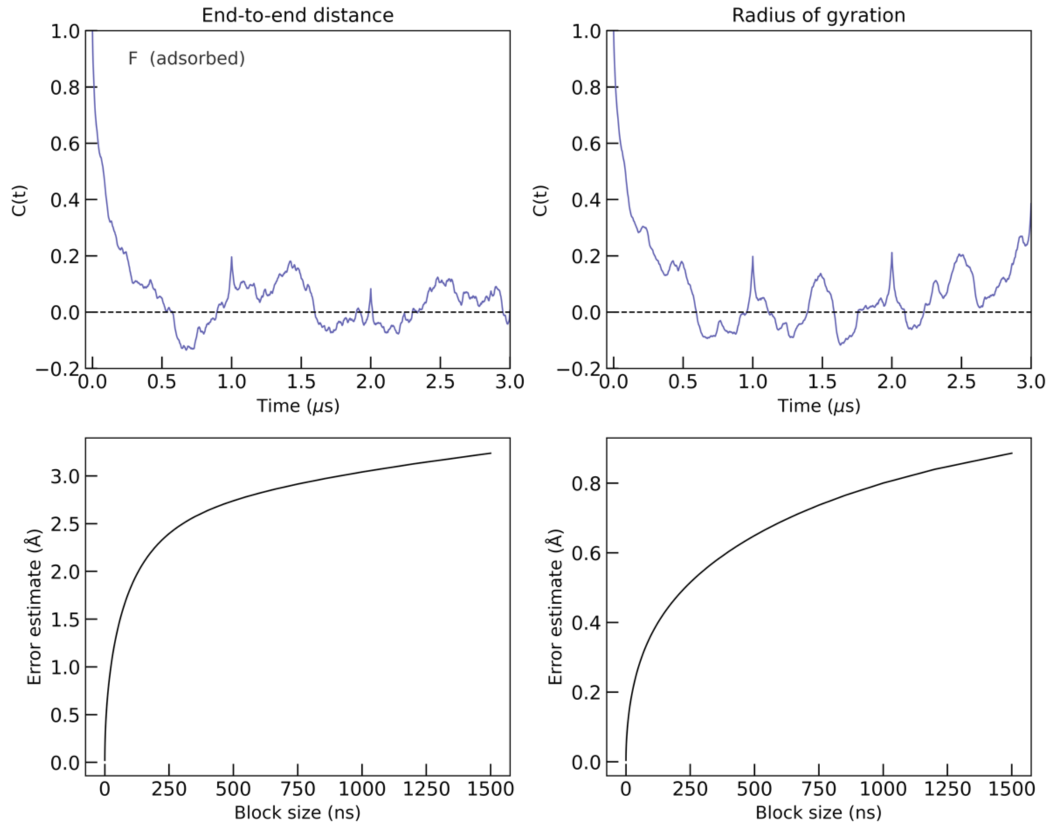

Supplementary Figure 40. Autocorrelation (top) and error estimates (bottom) from block averaging of the end-to-end distance (left) and radius of gyration (right) for the concatenated trajectory of KEIF, adsorbed to Laponite® with the force field/water model F.

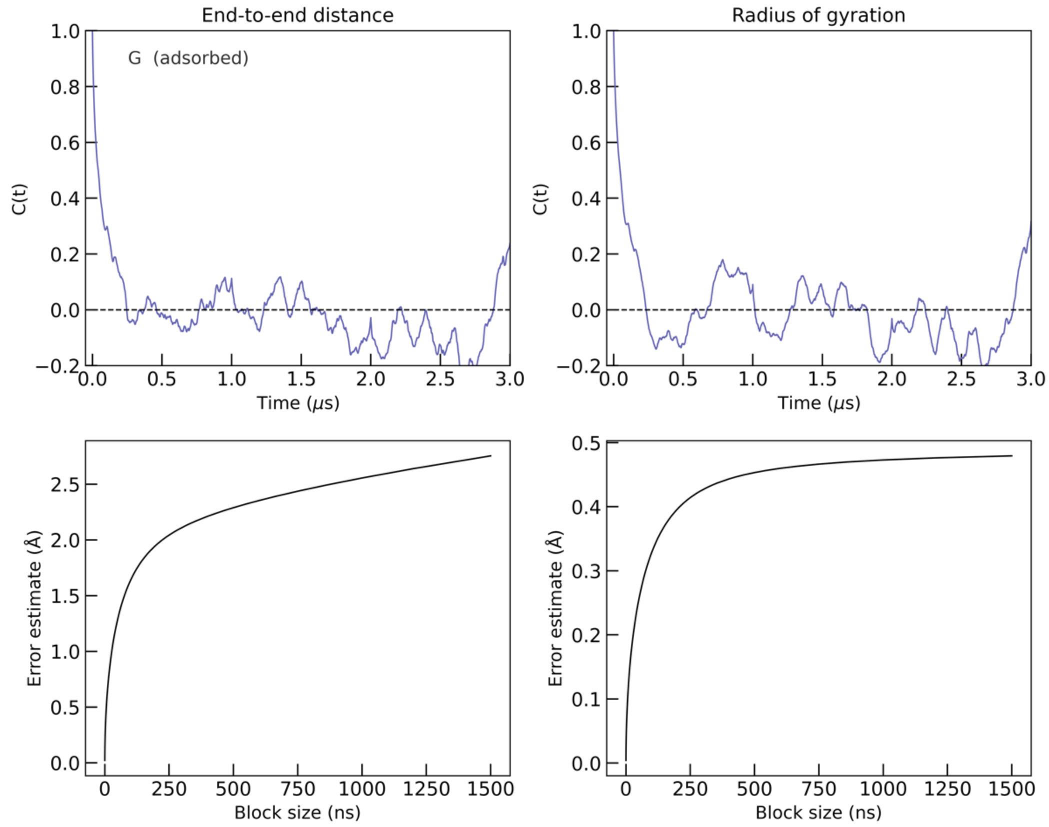

Supplementary Figure 41. Autocorrelation (top) and error estimates (bottom) from block averaging of the end-to-end distance (left) and radius of gyration (right) for the concatenated trajectory of KEIF, adsorbed to Laponite® with the force field/water model G.

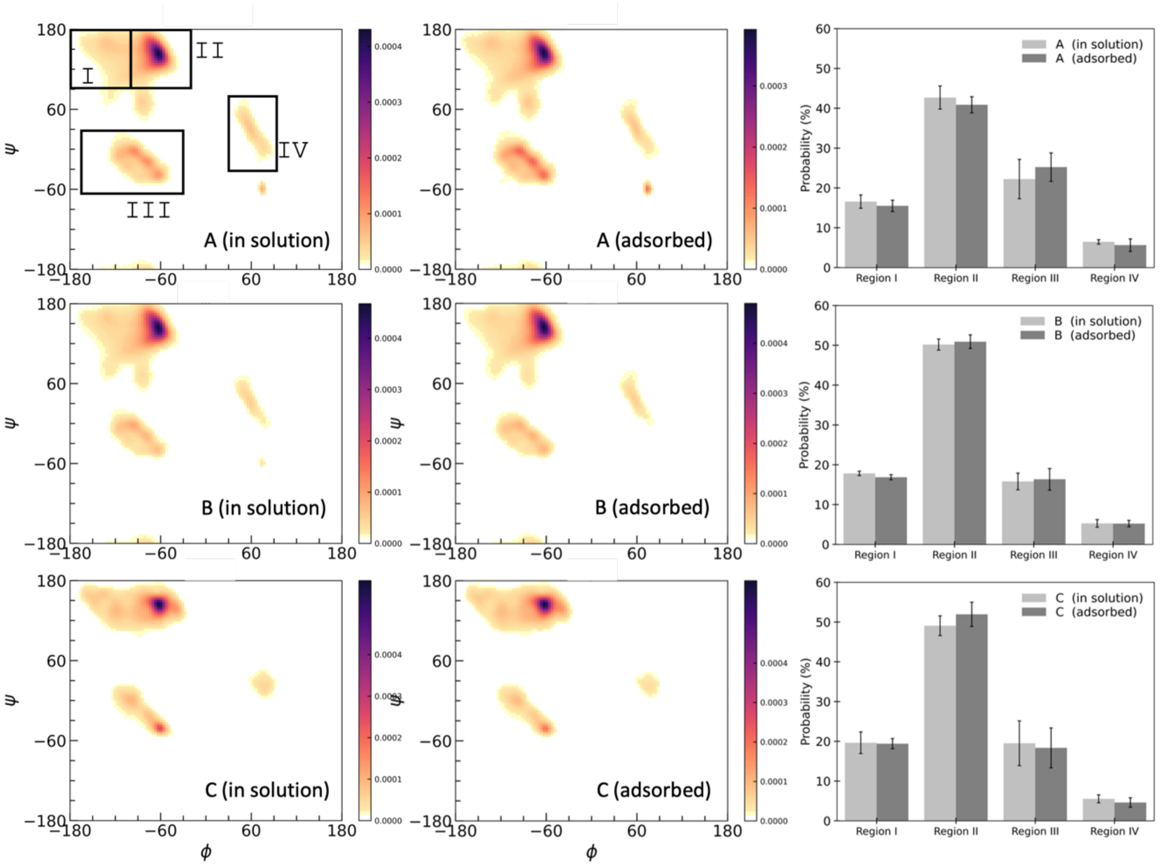

Supplementary Figure 42. Ramachandran plots for the concatenated trajectories of KEIF in solution (left) and adsorbed (center), as well as their integrated values in Regions I-IV, for the CHARMM based force fields/water models A, B, and C. Error bars represent the standard deviation between replicates.

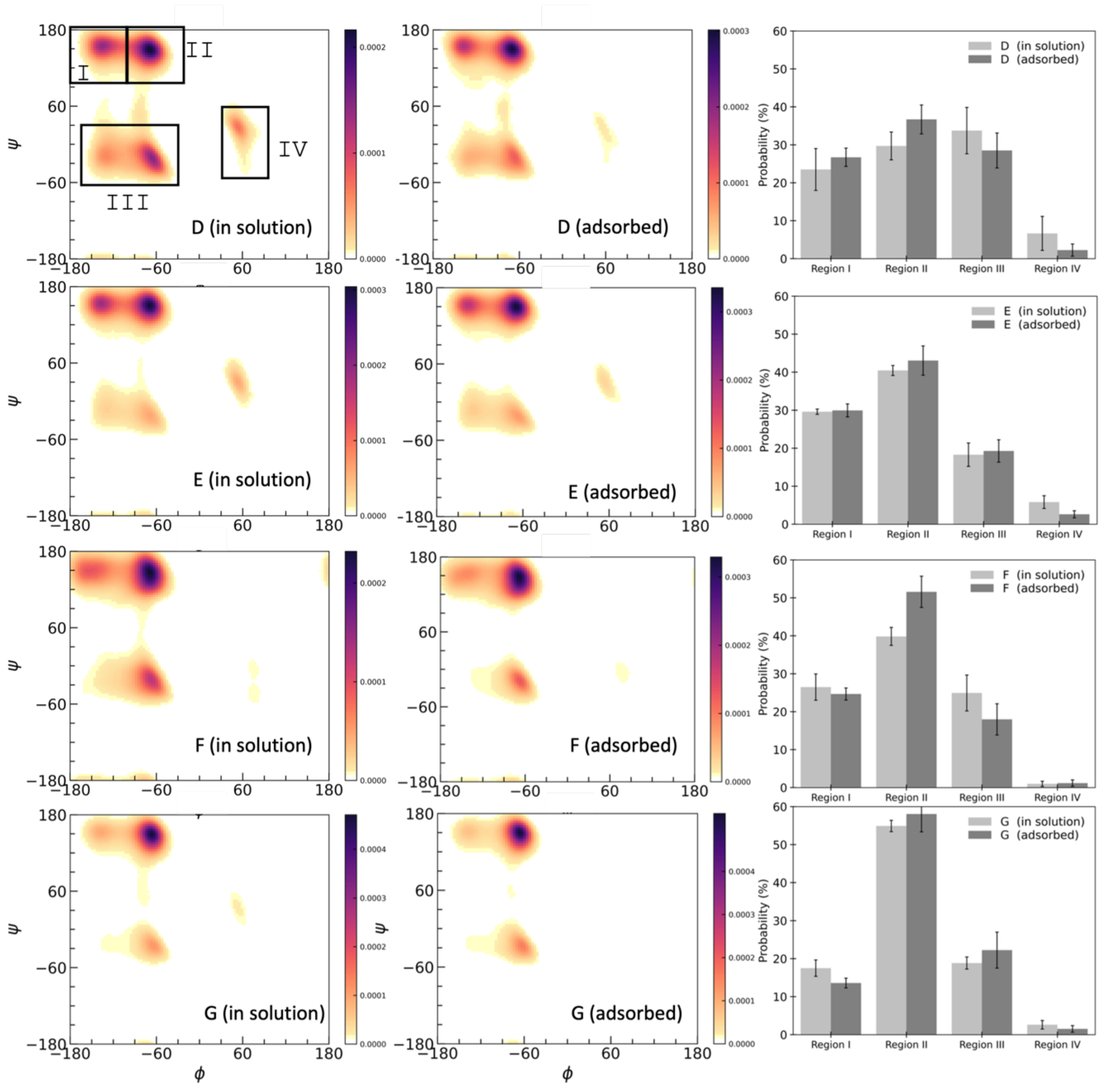

Supplementary Figure 43. Ramachandran plots for the concatenated trajectories of KEIF in solution (left) and adsorbed (center), as well as their integrated values in Regions I-IV, for the AMBER based force fields/water models D, E, F, and G. Error bars represent the standard deviation between replicates.

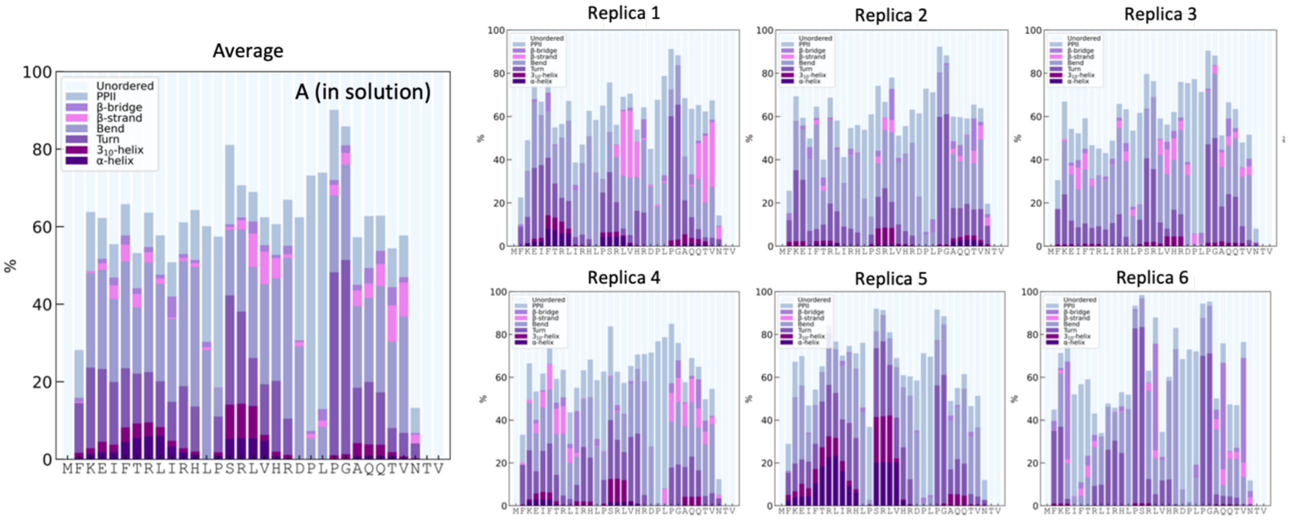

Supplementary Figure 44. Secondary structure content along the sequence from DSSPPII analysis of KEIF in solution, showing all six replicas and their average of fields/water model A.

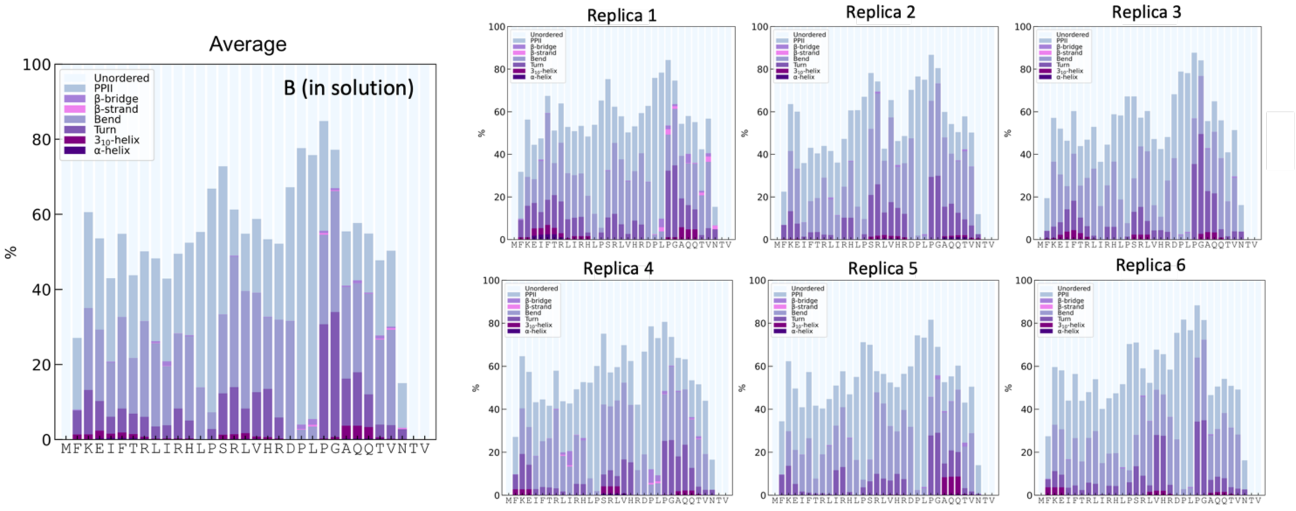

Supplementary Figure 45. Secondary structure content along the sequence from DSSPPII analysis of KEIF in solution, showing all six replicas and their average of fields/water model B.

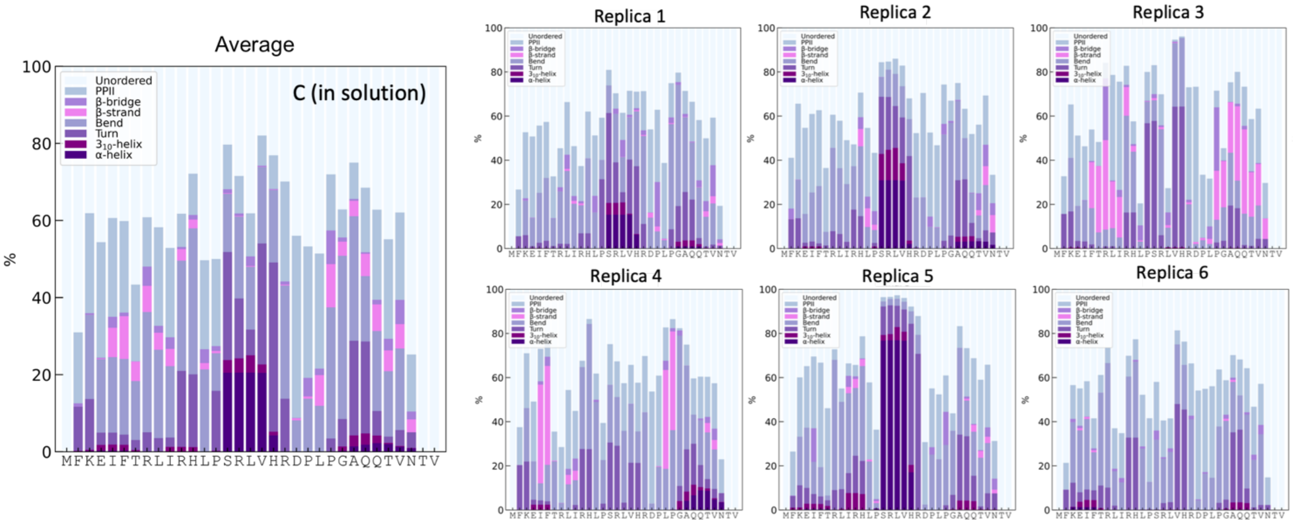

Supplementary Figure 46. Secondary structure content along the sequence from DSSPPII analysis of KEIF in solution, showing all six replicas and their average of fields/water model C.

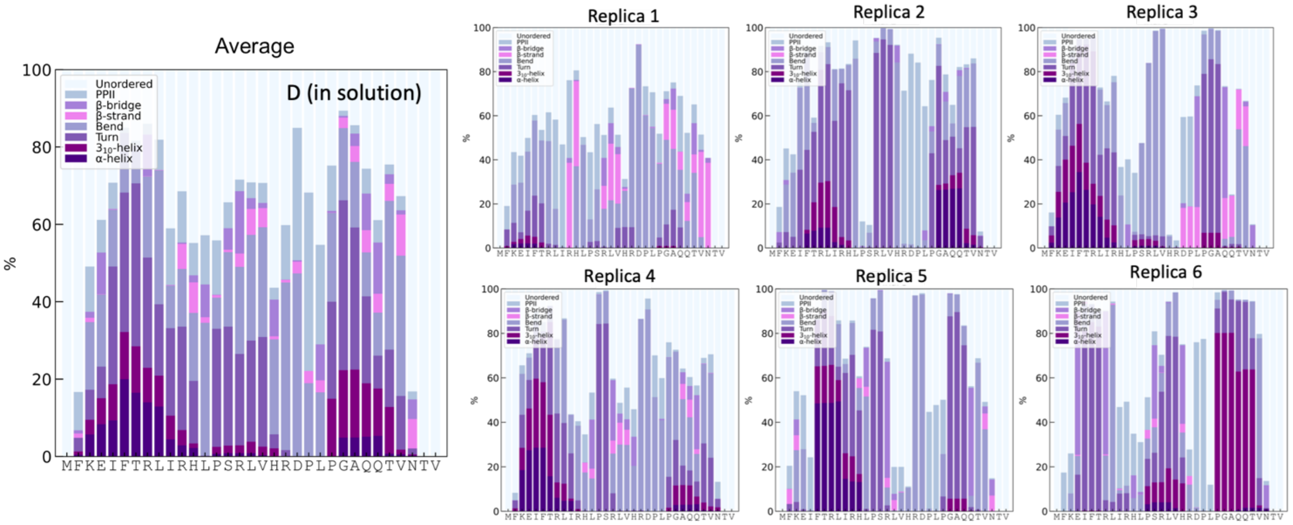

Supplementary Figure 47. Secondary structure content along the sequence from DSSPPII analysis of KEIF in solution, showing all six replicas and their average of fields/water model D.

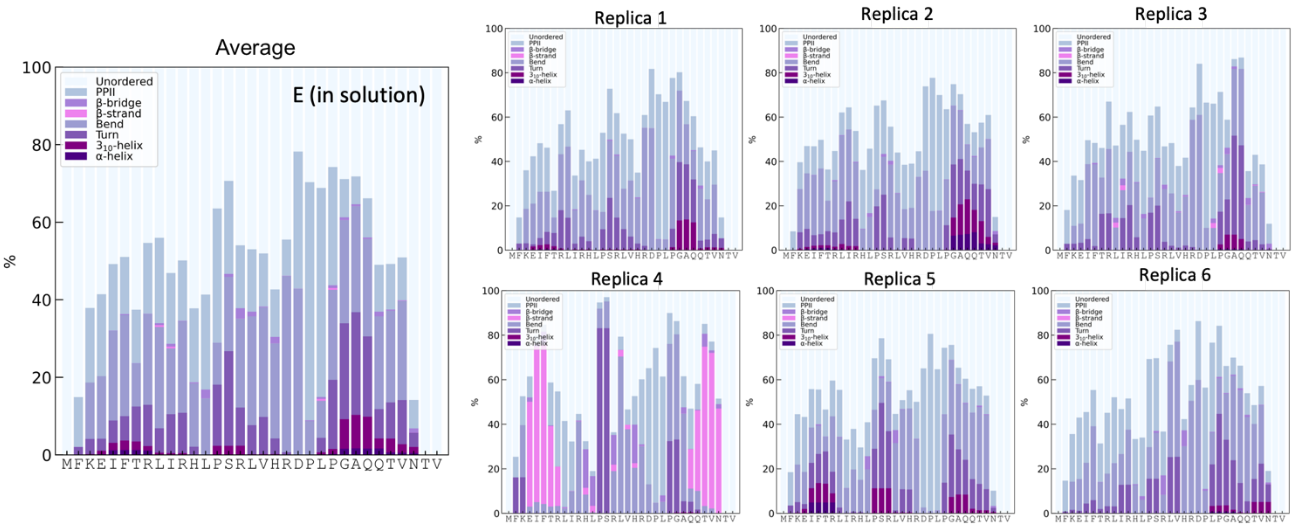

Supplementary Figure 48. Secondary structure content along the sequence from DSSPPII analysis of KEIF in solution, showing all six replicas and their average of fields/water model E.

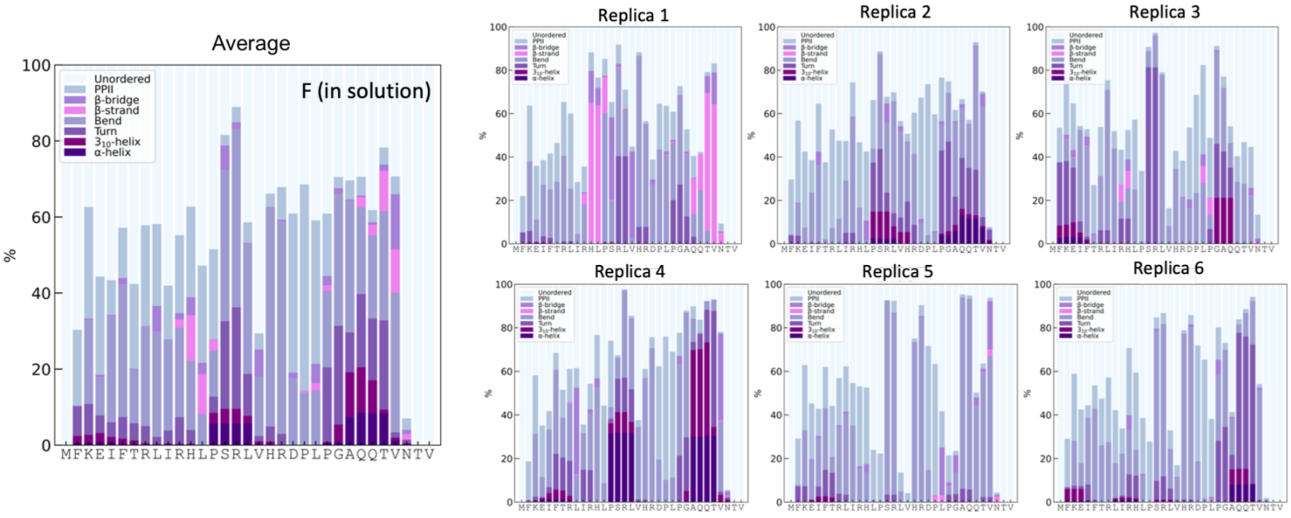

Supplementary Figure 49. Secondary structure content along the sequence from DSSPPII analysis of KEIF in solution, showing all six replicas and their average of fields/water model F.

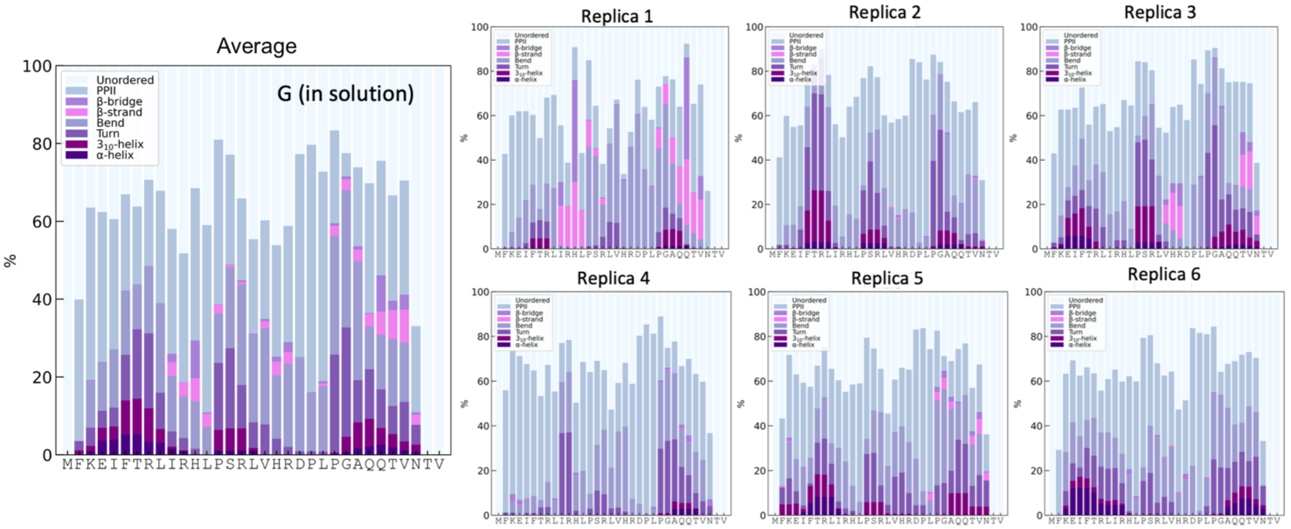

Supplementary Figure 50. Secondary structure content along the sequence from DSSPPII analysis of KEIF in solution, showing all six replicas and their average of fields/water model G.

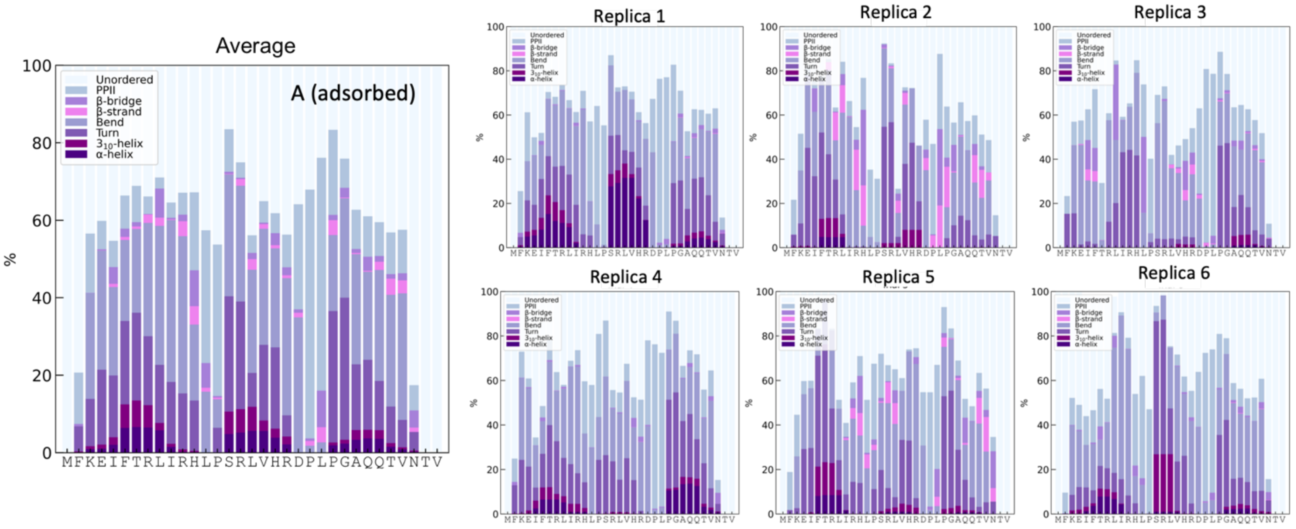

Supplementary Figure 51. Secondary structure content along the sequence from DSSPPII analysis of KEIF adsorbed to Laponite®, showing all six replicas and their average of fields/water model A.

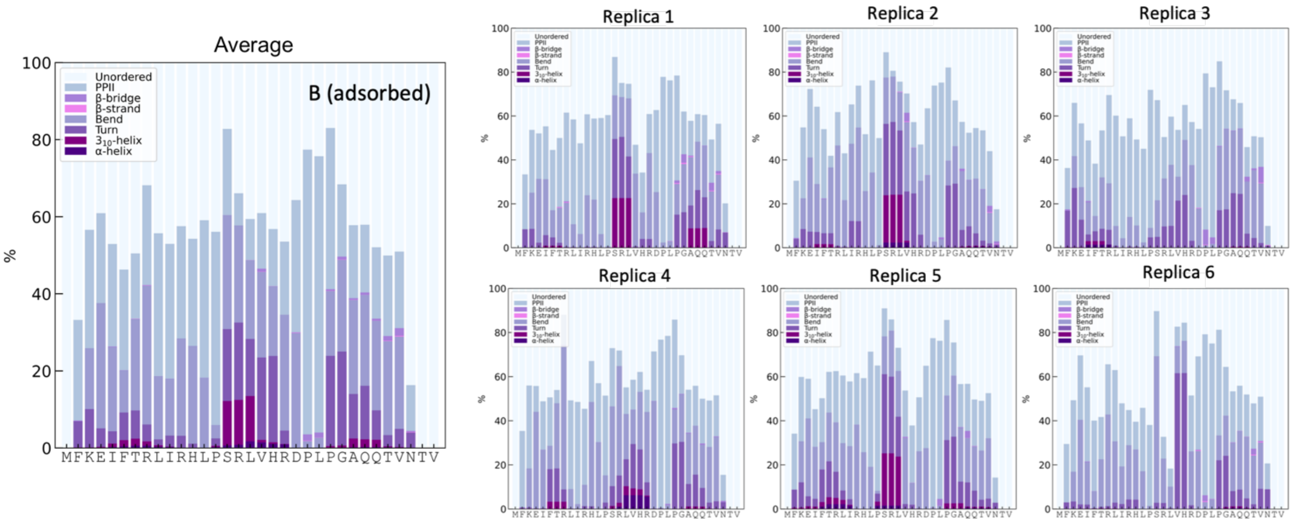

Supplementary Figure 52. Secondary structure content along the sequence from DSSPPII analysis of KEIF adsorbed to Laponite®, showing all six replicas and their average of fields/water model B.

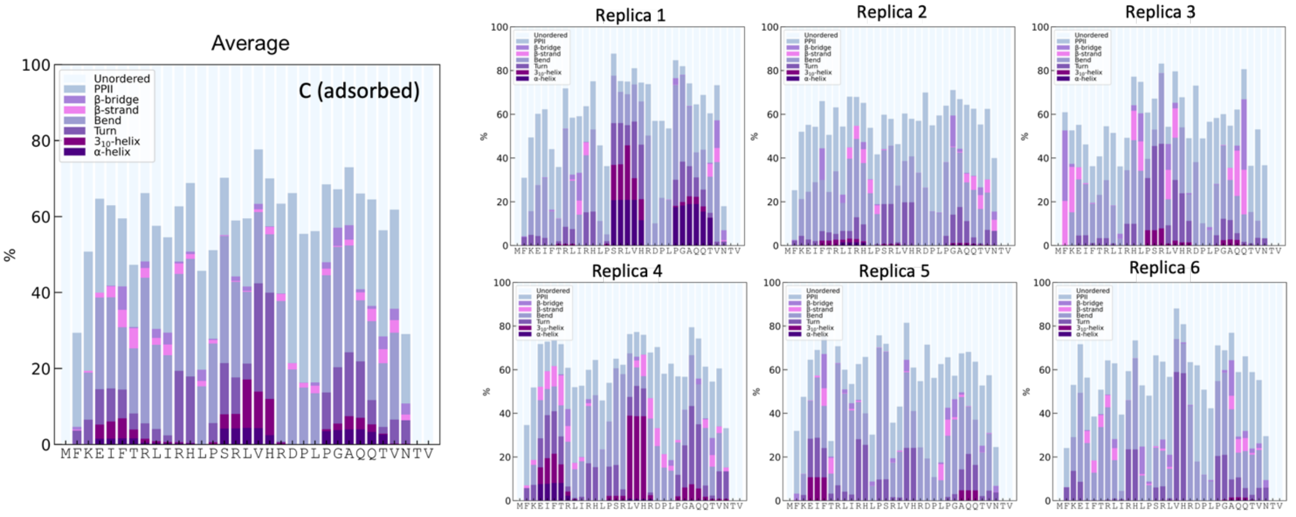

Supplementary Figure 53. Secondary structure content along the sequence from DSSPPII analysis of KEIF adsorbed to Laponite®, showing all six replicas and their average of fields/water model C.

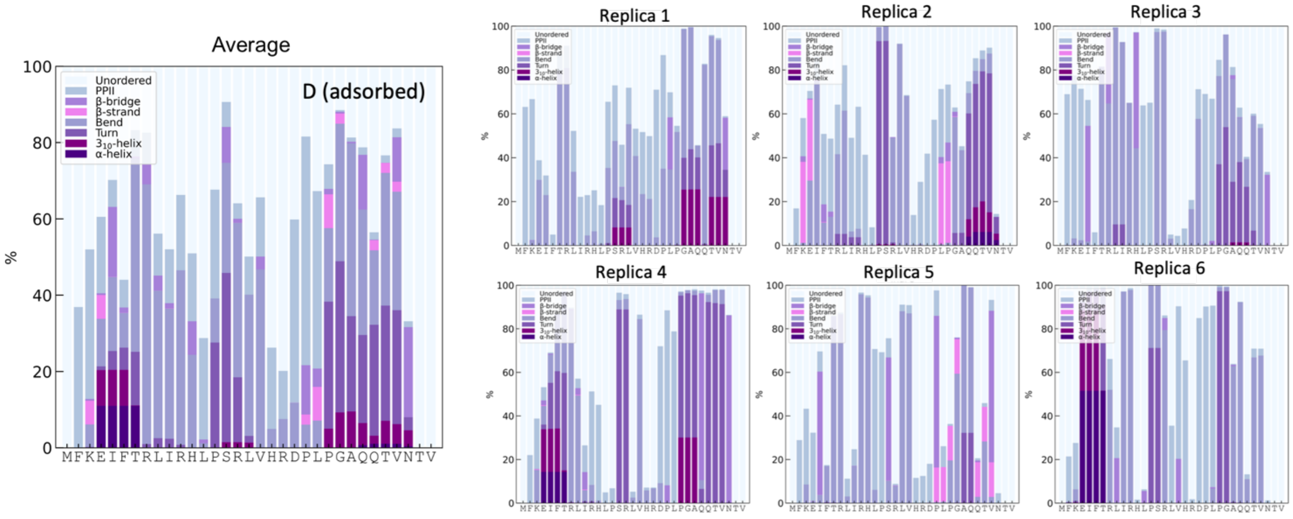

Supplementary Figure 54. Secondary structure content along the sequence from DSSPPII analysis of KEIF adsorbed to Laponite®, showing all six replicas and their average of fields/water model D.

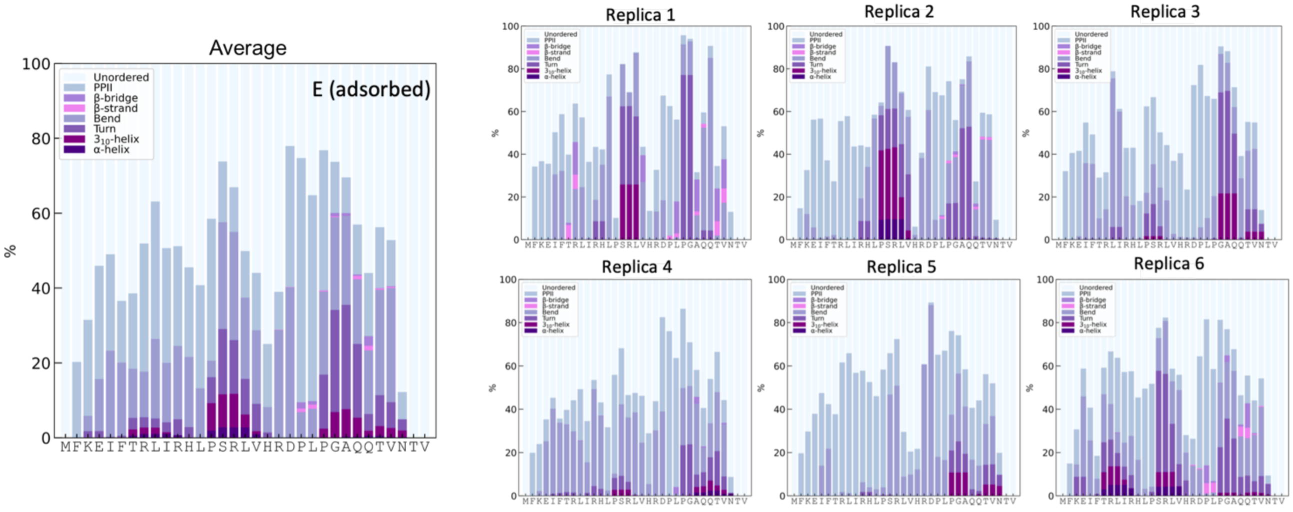

Supplementary Figure 55. Secondary structure content along the sequence from DSSPPII analysis of KEIF adsorbed to Laponite®, showing all six replicas and their average of fields/water model E.

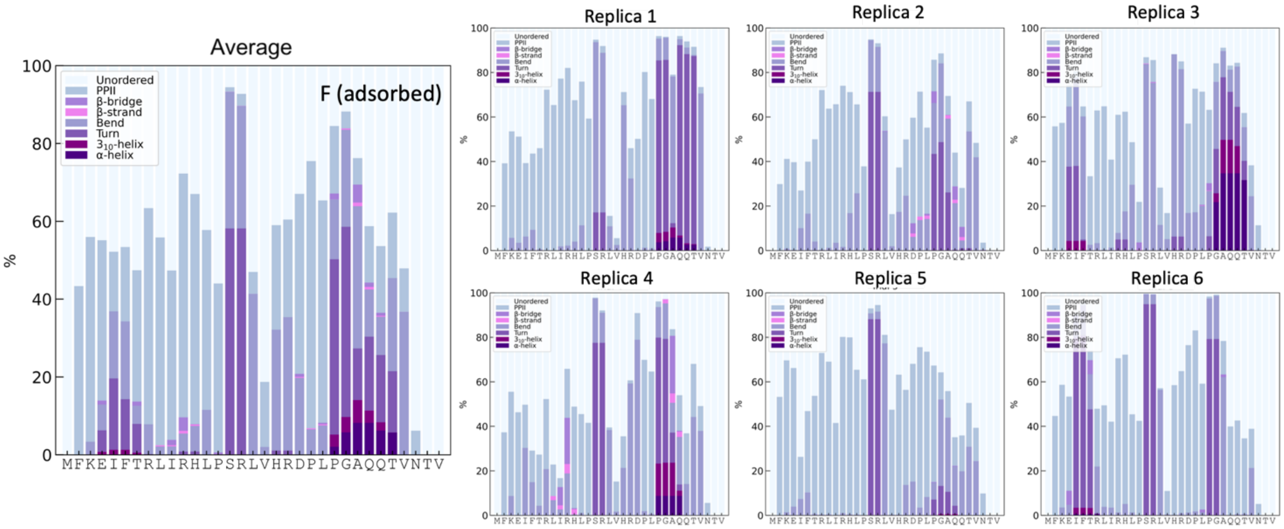

Supplementary Figure 56. Secondary structure content along the sequence from DSSPPII analysis of KEIF adsorbed to Laponite®, showing all six replicas and their average of fields/water model F.

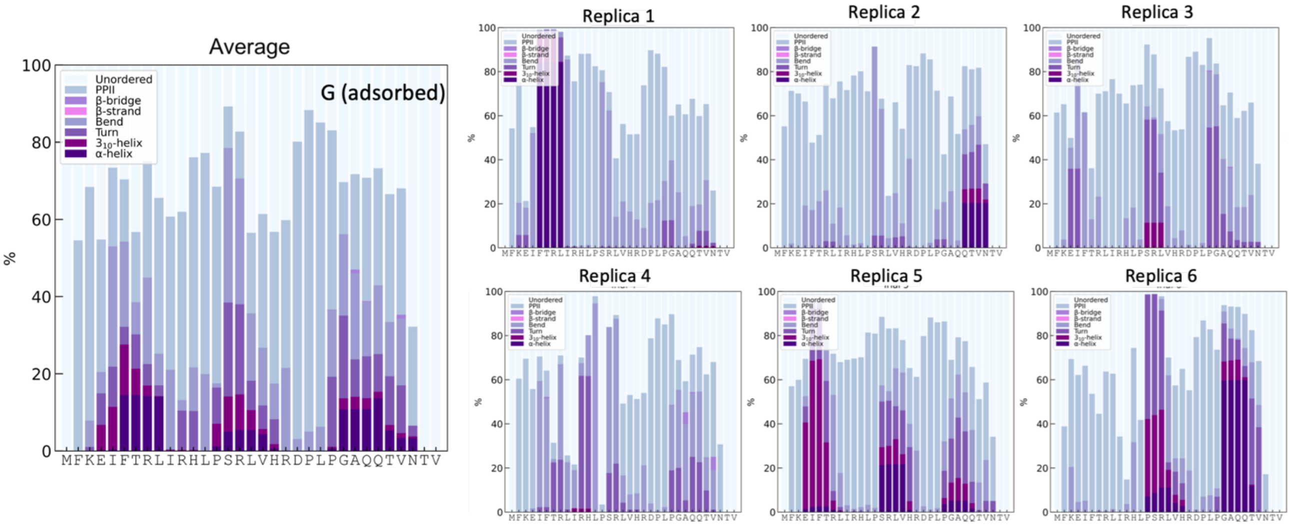

Supplementary Figure 57. Secondary structure content along the sequence from DSSPPII analysis of KEIF adsorbed to Laponite®, showing all six replicas and their average of fields/water model G.

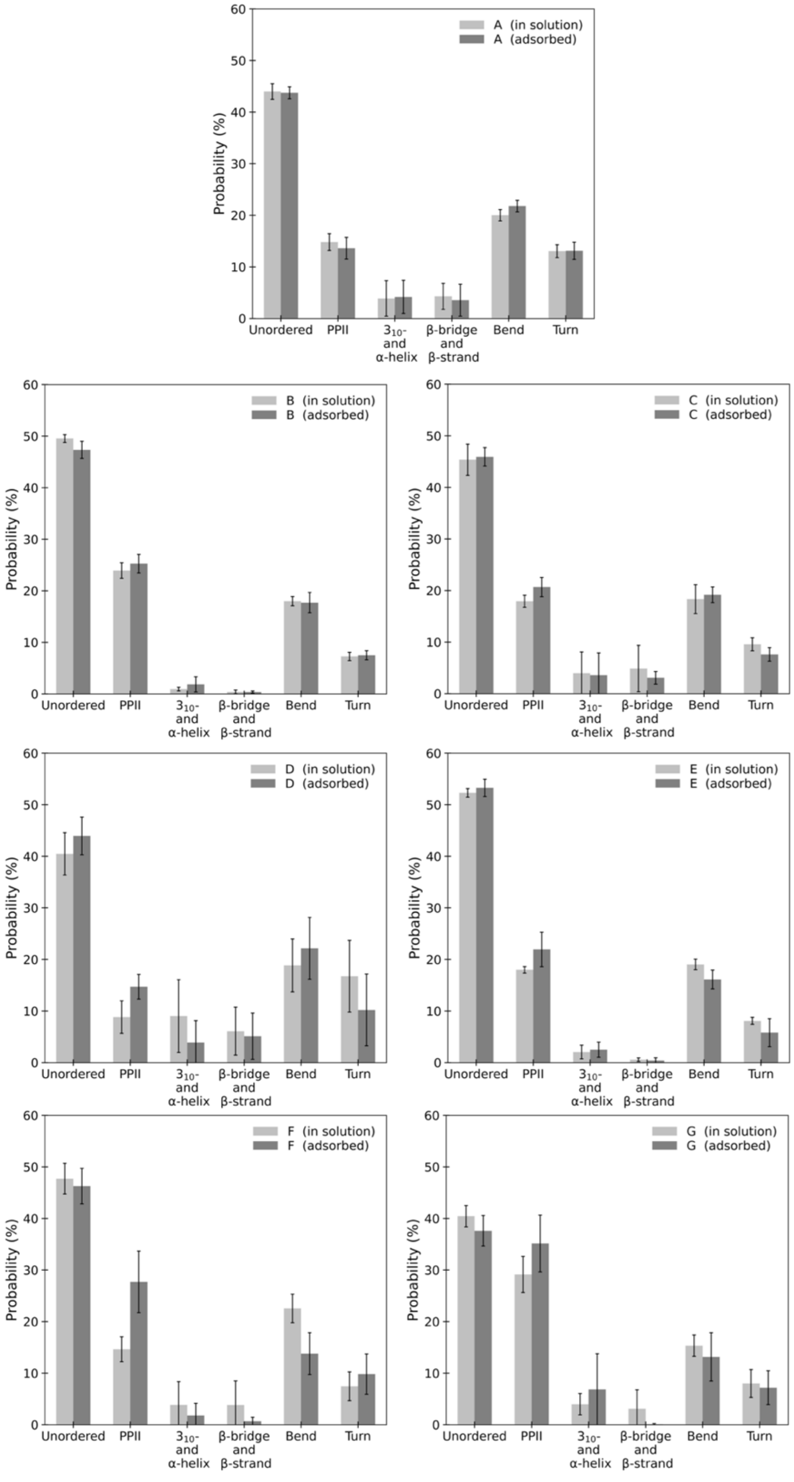

Supplementary Figure 58. Average secondary structure content from DSSPPII analysis of KEIF in solution and adsorbed to Laponite® for all seven force field/water models A, B, C, D, E, F, and G. Error bars represent the standard deviation between replicates.

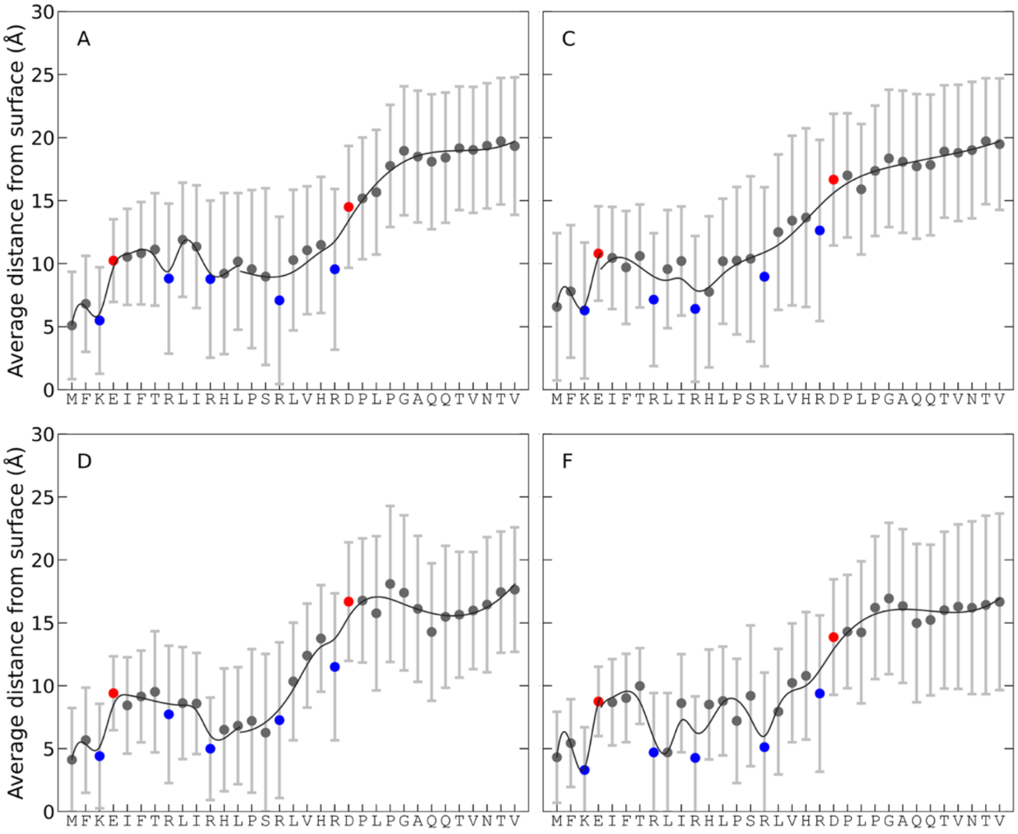

Supplementary Figure 59. The average distance from the Laponite surface for each residue and its standard deviation, calculated from the change in minimum distance between each pair over time for the concatenated trajectory of the replicates for the force field/water models A, C, D, and F. Charge of residues indicated with the colours: blue (positive), red (negative) and grey (neutral). Lines are included as a “guide to the eye”.

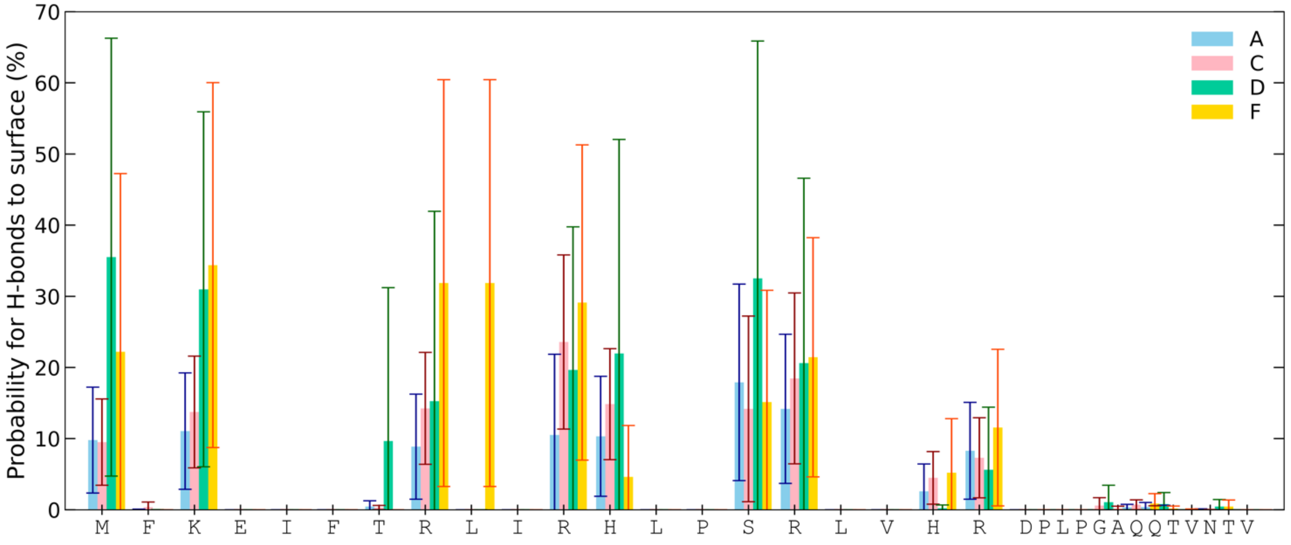

Supplementary Figure 60. The probability for each residue to hydrogen bond. Error bars represent the standard deviation between replicates. Results are shown for the force field/water models A, C, D, and F.

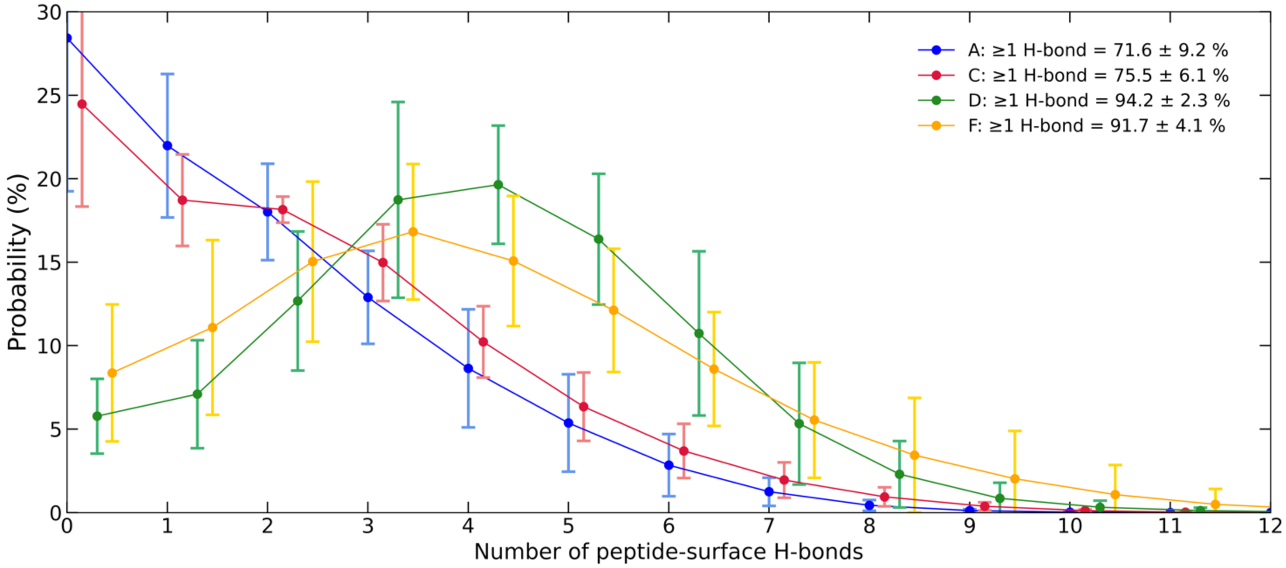

Supplementary Figure 61. The probability of multiple hydrogen bonds between KEIF and Laponite® during the simulations and the probability of at least one hydrogen bond is included in the legend. Results are shown for the force field/water models A, C, D, and F.

4 References

ARRONDO, J. L. R., MUGA, A., CASTRESANA, J. & GONI, F. M. 1993. Quantitative studies of the structure of proteins in solution by Fourier-transform infrared-spectroscopy. *Prog. Biophys. Mol. Biol.,* 59**,** 23-56.

CYGAN, R. T., LIANG, J. J. & KALINICHEV, A. G. 2004. Molecular models of hydroxide, oxyhydroxide, and clay phases and the development of a general force field. *J. Phys. Chem. B,* 108**,** 1255-1266.

MICSONAI, A., WIEN, F., BULYAKI, E., KUN, J., MOUSSONG, E., LEE, Y.-H., GOTO, Y., REFREGIERS, M. & KARDOS, J. 2018. BeStSel: a web server for accurate protein secondary structure prediction and fold recognition from the circular dichroism spectra. *Nucleic Acids Res. Spec. Publ.,* 46**,** W315-W322.

MICSONAI, A., WIEN, F., KERNYA, L., LEE, Y.-H., GOTO, Y., REFREGIERS, M. & KARDOS, J. 2015. Accurate secondary structure prediction and fold recognition for circular dichroism spectroscopy. *PNAS,* 112**,** E3095-E3103.

SINGH, B. R. 1999. Basic Aspects of the Technique and Applications of Infrared Spectroscopy of Peptides and Proteins. *Infrared Analysis of Peptides and Proteins.* American Chemical Society.
